# Proteomic Insights into Starvation of Nitrogen-Replete Cells of *Nostoc* sp. PCC 7120 under β-N-Methylamino-L-Alanine (BMAA) Treatment

**DOI:** 10.3390/toxins12060372

**Published:** 2020-06-04

**Authors:** Olga A. Koksharova, Ivan O. Butenko, Olga V. Pobeguts, Nina A. Safronova, Vadim M. Govorun

**Affiliations:** 1 Belozersky Institute of Physical-Chemical Biology, Lomonosov Moscow State University, Leninskie Gory, 1-40, 119992 Moscow, Russia; safronova.nina2007@mail.ru; 2 Institute of Molecular Genetics, Russian Academy of Sciences, Kurchatov Square, 2, 123182 Moscow, Russia; 3 Federal Research and Clinical Centre of Physical-Chemical Medicine, 119435 Moscow, Russia; ivan.butenko@gmail.com (I.O.B.); nikitishena@mail.ru (O.V.P.); vgovorun@yandex.ru (V.M.G.)

**Keywords:** *Anabaena*, nitrogen metabolism, GlnB (PII) protein, NtcA, 2-oxoglutarate, amino acids metabolism, carbon fixation, oxidative stress, RecA, thioredoxin I

## Abstract

All cyanobacteria produce a neurotoxic non-protein amino acid β-N-methylamino-L-alanine (BMAA). However, the biological function of BMAA in the regulation of cyanobacteria metabolism still remains undetermined. It is known that BMAA suppresses the formation of heterocysts in diazotrophic cyanobacteria under nitrogen starvation conditions, and BMAA induces the formation of heterocyst-like cells under nitrogen excess conditions, by causing the expression of heterocyst-specific genes that are usually “silent” under nitrogen-replete conditions, as if these bacteria receive a nitrogen deficiency intracellular molecular signal. In order to find out the molecular mechanisms underlying this unexpected BMAA effect, we studied the proteome of cyanobacterium *Nostoc* sp. PCC 7120 grown under BMAA treatment in nitrogen-replete medium. Experiments were performed in two experimental settings: (1) in control samples consisted of cells grown without the BMAA treatment and (2) the treated samples consisted of cells grown with addition of an aqueous solution of BMAA (20 µM). In total, 1567 different proteins of *Nostoc* sp. PCC 7120 were identified by LC-MS/MS spectrometry. Among them, 80 proteins belonging to different functional categories were chosen for further functional analysis and interpretation of obtained proteomic data. Here, we provide the evidence that a pleiotropic regulatory effect of BMAA on the proteome of cyanobacterium was largely different under conditions of nitrogen-excess compared to its effect under nitrogen starvation conditions (that was studied in our previous work). The most significant difference in proteome expression between the BMAA-treated and untreated samples under different growth conditions was detected in key regulatory protein PII (GlnB). BMAA downregulates protein PII in nitrogen-starved cells and upregulates this protein in nitrogen-replete conditions. PII protein is a key signal transduction protein and the change in its regulation leads to the change of many other regulatory proteins, including different transcriptional factors, enzymes and transporters. Complex changes in key metabolic and regulatory proteins (RbcL, RbcS, Rca, CmpA, GltS, NodM, thioredoxin 1, RpbD, ClpP, MinD, RecA, etc.), detected in this experimental study, could be a reason for the appearance of the “starvation” state in nitrogen-replete conditions in the presence of BMAA. In addition, 15 proteins identified in this study are encoded by genes, which are under the control of NtcA—a global transcriptional regulator—one of the main protein partners and transcriptional regulators of PII protein. Thereby, this proteomic study gives a possible explanation of cyanobacterium starvation under nitrogen-replete conditions and BMAA treatment. It allows to take a closer look at the regulation of cyanobacteria metabolism affected by this cyanotoxin.

## 1. Introduction

Cyanobacteria are unique microorganisms capable of oxygenic photosynthesis. Some cyanobacteria are able to fix atmospheric nitrogen in anaerobic conditions using heterocysts. These specialized cells are formed at semi-regular intervals in the filaments under nitrogen starvation conditions, when nitrogen is limited in the environment. In a wide variety of water and land ecosystems, cyanobacteria are advantageous symbionts of plants and marine animals and essential producers of carbon and nitrogen [1]. Cyanobacteria also produce many different metabolites, among them are dangerous cyanotoxins that may have physiological and ecological impacts on their producers’ life, as well as on natural cyanobacterial communities [2]. Investigating the biological functions of cyanotoxins is an important and challenging scientific task [3,4,5]. Beta-N-methylamino-L-alanine (BMAA) is an insufficiently studied cyanotoxin, which can be produced on a wide range of concentrations—from 0.001–0.3 to approximately 1000–6000 μg g^−1^ dry weight—by all studied cyanobacteria [6] and diatoms [7] that often live in the same natural environment [8]. BMAA bioaccumulates in plants and animals [6,9,10,11,12]. Consequently, high amounts of BMAA may enter human bodies via bioaccumulation in seeds and seafood [6,9,10,11,12]. This causes concern due to the subsequent development of human neurodegenerative diseases, such as amyotrophic lateral sclerosis and parkinsonism-dementia [13,14]. For that reason, BMAA effects were studied, so far mostly in mammalian models, for review [15]. Knowledge on the synthesis mechanisms of this neurotoxic non-protein amino acid and on the functional role of BMAA in cyanobacteria cells is still scant [16,17,18,19,20,21,22,23]. There are a few experimental studies dedicated to the notable and specific biological impact of BMAA on nitrogen-fixing filamentous cyanobacterium *Nostoc* (also known as *Anabaena*) sp. PCC 7120 [20,21,22,23], which is known to be a favorable model organism for nitrogen fixation and heterocyst differentiation studies [24]. Its filaments contain only vegetative cells in the presence of combined nitrogen in the form of ammonium (NH_4_^+^) or nitrate (NO_3−_) ions. When nitrogen is removed from the growth medium, some vegetative cells turn into heterocysts to protect nitrogenase (a nitrogen fixation enzyme) from oxygen and to provide neighboring vegetative cells with nitrogenous compounds [24]. Heterocyst development is a well-regulated complex process [1,24]. Studies on *Nostoc* sp. PCC 7120 have shown that BMAA strongly and specifically inhibits the nitrogenase enzyme activity in mature heterocysts [20,21], as well as gene expression of *nifH* [21], which encodes nitrogenase reductase—one of the main components of nitrogenase. If this non-protein amino acid is added to cyanobacteria cells at the beginning of starvation, the heterocyst formation is completely inhibited by BMAA. Moreover, the transcription of the key heterocyst-specific genes, *hetR* and *hepA*, is downregulated during nitrogen deprivation in *Nostoc* sp. PCC 7120 [21]. Recently, we demonstrated experimentally, by using a proteomic approach, that under nitrogen starvation, the BMAA addition downregulates the key nitrogen regulatory protein PII (GlnB), glutamyl-tRNA synthetase (*gltX*) enzyme and different proteins involved in nitrogen metabolism and heterocyst formation, as well as proteins involved in carbon fixation and photosynthesis in cyanobacteria cells [23].

We have found one more unexpected regulatory effect of BMAA on *Nostoc* sp. PCC 7120 cells that were grown in nitrogen-replete medium [22]. In the presence of nitrogen, the addition of this cyanotoxin leads to the formation of heterocyst-like cells and to the activation of heterocyst-specific gene expression [22]. Normally, in *Nostoc* sp. PCC 7120 cells, these genes are “silent” under nitrogen-replete conditions. Therefore, it could be assumed that in the conditions of nitrogen excess, BMAA induces a nitrogen deficiency signal(s) that can activate heterocyst-specific genes. However, possible reasons for such regulatory changes in cyanobacteria development are unknown to date. To examine the molecular mechanisms underlying this phenomenon, we used the experimental proteomic approach. Proteomic analysis allows us to take a deeper look into the functional changes that occurred in cyanobacteria cells after BMAA is added into nitrogen-replete growth medium.

The goal of this work was to study the regulatory effect of exogenous BMAA on the proteome of *Nostoc* sp. PCC 7120 (herein referred to as *Nostoc*) in nitrogen-replete conditions.

## 2. Results and Discussion

### 2.1. Proteins Affected by BMAA Under Nitrogen-Replete Conditions

In order to test BMAA effect on *Nostoc* under nitrogen-replete conditions, cyanobacterium was grown in three independent biological replicates in sodium nitrate containing BG11N medium for 48 h in two experimental settings: (1) control samples consisted of cells grown without the BMAA treatment and (2) the treated samples consisted of cells grown with addition of an aqueous solution of BMAA (20 µM), as it was performed earlier [22,23]. The analysis of cyanobacteria samples by using LC-MS/MS methods resulted in the identification of 1567 different proteins of *Nostoc* (Supplementary Table S1). Among them, 80 proteins belonging to different functional categories have been chosen for further functional analysis based on the statistical significance of the observed differences between the BMAA-treated samples and control samples (Table 1 and Table 2 and Supplementary Table S2).

Differentially regulated proteins are presented in Table 2 and Supplementary Table S2. Each table contains the following information: the name of the identified protein, corresponding gene number, metabolic pathway or function (with the exception of hypothetical proteins), fold change between BMAA-treated and control samples and *p*-value. BMAA affects proteins with diverse functions within different metabolic pathways in cyanobacteria cells. Among them are proteins that are involved in nitrogen metabolism, carbon fixation, amino acid synthesis, fatty acid biosynthesis, sulfur metabolism, starch and sucrose metabolism, stress response and DNA repair, cell division, regulatory proteins and proteases (Table 1). Fourteen proteins were shown to be more than two-fold downregulated in BMAA-exposed cells. Thirteen proteins were more than two-fold upregulated in BMAA-treated cells (Table 1 and Table 2 and Supplementary Table S2). Eighteen of the identified proteins are considered as “hypothetical” proteins (Table 1 and Supplementary Table S2).

A discussion of the selected proteins specified by their functional category and their roles in cyanobacteria cells can be found bellow in Section 2.2, Section 2.3, Section 2.4, Section 2.5, Section 2.6, Section 2.7 and Section 2.8.

### 2.2. Nitrogen Status Sensing and Nitrogen Assimilation 

**Figure 1:**
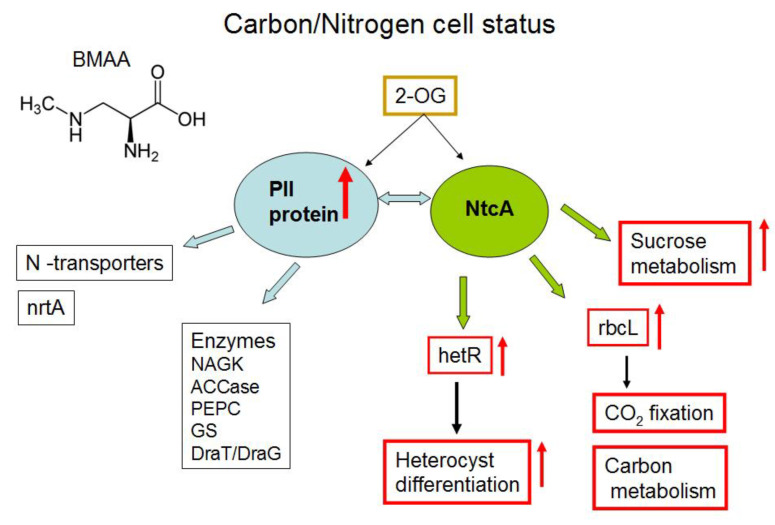
Graphical representation of main targets of key regulatory protein PII under nitrogen-replete cells of *Nostoc*. Light-blue arrows represent interactions between protein PII and its main protein partners [28]. Red arrows indicate upregulation (↑) of the rbcL protein, upregulation of the gene *hetR* expression and influence on cellular processes that were identified in this study and in our previous work [22]. Green arrows indicate NtcA regulation, and black arrows show involvement of *hetR* and rbcL in heterocyst differentiation and CO_2_ fixation, respectively.

Cyanobacteria can maintain their cellular homeostasis by sensing and regulating the intracellular carbon/nitrogen (C/N) balance. They possess a finely regulated signal transduction network [24,25,26,27,28,29,30,31], which involves the PII-signaling protein, metabolite 2 oxoglutarate (2-OG), NtcA transcription factor (Figure 1) and some other proteins. BMAA upregulated the key nitrogen regulatory protein PII (GlnB) (*all2319*) (Table 2) in nitrogen-replete growth conditions. PII protein was upregulated almost two-fold in BMAA-treated samples. The sensor-transducer protein PII plays a main role in the control of nitrogen metabolism in bacteria [25]. It transforms signals of carbon, nitrogen and energy deficiency or abundance into changes in the activities of regulatory proteins and gene expression, channels and enzymes activities [26,27,28] (Figure 1).

**Figure 2:**
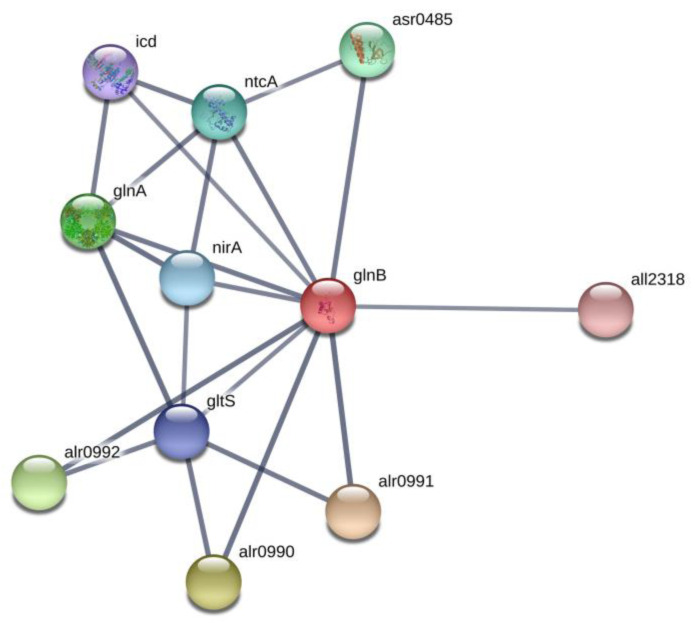
Network of PII (GlnB) and its protein partners according to STRING (https://string-db.org). In this figure: proteins alr0990, alr0991, alr0992 are ammonium transporters, gltS is glutamate synthase, glnA is glutamine synthetase, nirA is ferredoxin-nitrite reductase, ntcA is a global nitrogen regulator and a transcriptional activator of genes, which are subjected to nitrogen control, icd is an isocitrate dehydrogenase, asr0485 is a PII interaction protein X and all2318 is the RNA-binding protein TAB2.

The PII signal-transduction protein plays a key role in the control of various nitrogen-related transporters, enzymes and transcription factors (Figure 1 and Figure 2) [28]. In our previous proteomic study [23], we found that BMAA downregulated the PII protein in nitrogen-starved *Nostoc* cells that should have formed heterocysts, however, this process is repressed by BMAA [21]. Remarkably, in nitrogen starvation conditions, the addition of BMAA alters the expression of the nitrate transporter nrtA (*alr0608*) [23], a partner of PII protein [28,29]. PII protein negatively regulates nitrate transporters [28]. In our proteomic study [23], upregulation of nrtA protein was observed at the same time as downregulation of PII under BMAA treatment of nitrogen-starved *Nostoc* cells [23]. In the present study, PII protein was upregulated (Table 2, Figure 1) and one would presume that nitrate transporters could be downregulated. In this case, despite the presence of nitrate in growth medium, *Nostoc* cells probably cannot sufficiently uptake nitrate and therefore start to starve. Nitrogen starvation can trigger transcription of heterocyst-specific genes *hetR, hepA* and *nifH* and it leads to heterocyst-like cell formation [22]. This assumption ought to be tested in future experiments.

As we have shown in Reference [23], BMAA influenced the key regulatory protein PII and some of its functional protein partners. Taking into account that the global transcriptional regulator NtcA is one of the most important partners of PII [28,29,30] (Figure 1 and Figure 2), it could be proposed that one of the possible reasons for the enhancing effect of BMAA on *hetR* and *hepA* gene expression under repressive conditions in nitrogen-replete medium [22] is the upregulation of PII protein due to BMAA treatment and due to the expected subsequent changes in NtcA protein activity. In turn, this protein is the master regulator of genetic response for the C-to-N balance at the transcriptional level in cyanobacteria [31,32]. NtcA is a global regulatory transcription factor that directly regulates the expression of multiple genes, which are required not only for nitrogen and carbon assimilation but are also involved in a number of other metabolic pathways, such as DNA metabolism, transcription and translation, and central metabolism [32]. In this study, for example, we found that BMAA upregulates the protein RbcL (*alr1524*) (Table 2, Figure 1), which is also under NtcA control. Moreover, proteomic analysis revealed 15 proteins that are encoded by genes, which are under the transcriptional control of the global nitrogen regulator NtcA. Among them, 8 proteins were upregulated in the presence of BMAA: *all2319* (GlnB,PII), *alr1524* (RbcL), *alr1533* (Rubisco activase), *alr1554* (ABC transporter), *all2521* (cysteine synthase) and three hypothetical proteins (*alr4505, all1411, asr1156*), and 7 proteins were downregulated at this cyanotoxin’s presence: *alr1526* (RbcS), *all0089* (YggE), *all0129* (DNA-binding response regulator, OmpR family), *all4193* (S13), *alr2811* (valine-pyruvate aminotransferase), *all4613* (ilvG) and *all3570* (inorganic pyrophosphatase) (Table 2 and Supplementary Table S2). In our previous proteomic study [23], we found 17 proteins affected by BMAA that are encoded by genes, which are subjected to the transcription factor NtcA control. Comparing the two sets of NtcA-controlled proteins, obtained in the current and in the previous studies, we noticed that two key proteins—PII and RbcL—were regulated in opposite directions in nitrogen-replete and nitrogen-starving conditions, and that two hypothetical proteins (*alr4505* and *all1411*) were similarly highly upregulated in both conditions under BMAA treatment.

Glutamate synthase (GltS, *alr4344*) of *Nostoc* was downregulated under BMAA treatment in nitrogen-replete medium (Table 2, Figure 2, and Supplementary Figures S1 and S2). GltS [EC:1.4.7.1] is a complex iron-sulfur flavoprotein, which plays a key role in the ammonia assimilation pathways [33] and it is involved in glyoxylate and dicarboxylate metabolism (Supplementary Figure S2). GltS catalyzes a conversion, in which nitrogen from the glutamine side chain is transferred to 2-OG to form two molecules of glutamate. We propose that downregulation of GltS can lead to possible accumulation of 2-OG, a key regulatory metabolite [34] and, in turn, this affects PII and NtcA protein regulation. By the means of these proteins, this accumulation can affect the entire regulatory cascade of the cell. Note that high 2-OG levels are indicative of nitrogen deficiency in cyanobacteria [35]. It is of great interest to monitor in vivo the 2-oxoglutarate quantity dynamic in cyanobacteria. For example, biosensor-based on the fluorescence resonance energy transfer (FRET) microscopy techniques [36] could be applied for in vivo measurements of 2-OG dynamics at the cellular level under BMAA treatment.

### 2.3. CO_2_ Fixation and Carbon Dioxide-Concentrating Mechanism

In cyanobacteria, Ribulose-1,5-bisphosphate carboxylase/oxygenase (RubisCO) is the key CO_2_ fixation enzyme. In cyanobacteria cells, efficient carbon fixation by RubisCO is based on the ability to concentrate inorganic carbon (Ci) near the RubisCO active site. These phototrophic prokaryotes possess CO_2_-concentrating mechanisms (CCMs) to promote carbon fixation by the RubisCO. The signal molecule 2-OG is used by cyanobacteria as the cell C/N balance reporter, in such a way that under low nitrogen conditions (high 2-OG levels), the CCM would be diminished. This fact explains the reduced CO_2_ fixation rate under nitrogen-deprived conditions [27] (Figure 1). In the current study, we can see that BMAA disturbed CO_2_ fixation in cyanobacteria cells in the presence of combined nitrogen. Three proteins involved in CO_2_ fixation and the carbon dioxide-concentrating mechanism were upregulated by adding BMAA to nitrogen-replete cells of *Nostoc* (Table 2). The mentioned proteins are: RbcL (a large subunit of ribulose bisophosphate carboxylase (EC:4.1.1.39, gene *alr1524*) (Supplementary Figure S3), RuBisCO activase (*alr1533*) and CcmK protein (*all0868*), which is involved in the carbon dioxide-concentrating mechanism [37]. According to STRING, the CcmK protein interacts with the Ribulose bisphosphate carboxylase large chain (RbcL), with the Ribulose bisphosphate carboxylase small chain (RbcS) and with three proteins of the carbon dioxide-concentrating mechanism (ccmM, ccmN and ccmL) (Supplementary Figure S4).

**Figure 3:**
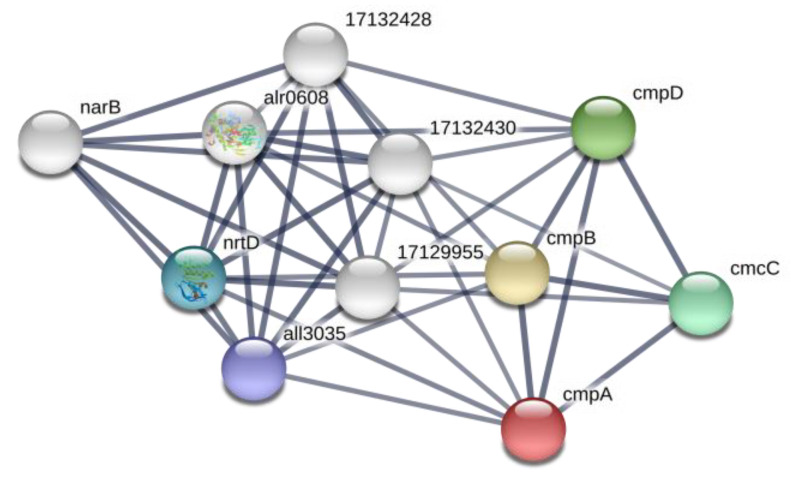
Network of cmpA (red ball) and its protein partners according to STRING (https://string-db.org). In this figure: proteins cmpB, cmpC, cmcD are bicarbonate transport ATP-binding proteins, all3035 is a nitrate/nitrite transport system ATP-binding protein (EC:3.6.3.-), nrtD is nitrate transport ATP-binding protein (*alr0611*), narB is nitrate reductase (*alr0612*), alr0608 is a nitrate/nitrite binding protein NrtA, 17129955 is nitrate transport permease protein NrtB (*alr0609*), 17132428 is nrtA nitrate-binding protein (*all3333*) and 17132430 is ABC-type nitrate transport permease protein NrtB (*all3335*).

At the same time, a bicarbonate transport bicarbonate-binding protein, CmpA (*alr2877*), was absent under treatment with BMAA (Table 2). This bicarbonate transport protein is a part of the ATP-binding Cassette (ABC) transport system CmpABCD in cyanobacteria. CmpA is highly homologous to the nitrate transport protein NrtA [38] and functionally interconnected with nitrate/nitrite transporters (Figure 3). Absence of CmpA may lead to the disturbance of carbon and nitrogen transport systems, and in this case, despite the presence of nitrate in growth medium, *Nostoc* cells ought to starve and this starvation signal may induce heterocyst-specific gene transcription that, in turn, results in heterocyst-like cell formation [22].

Two other proteins—the Ribulose bisphosphate carboxylase small chain (RbcS) (*alr1526*) and the CcmK protein that is involved in carbon dioxide-concentrating mechanism but encoded by another gene *alr0318*—were downregulated at BMAA presence (Table 2). Note that there are two gene clusters encoding ccmK proteins present in the *Nostoc* 7120 genome (https://www.genome.jp/kegg-bin/show_genomemap?ORG=ana&CHR=c&START_POS=960001). In our study, we found that CcmK proteins, which are encoded by genes (*all0868* and *alr0318*) from different gene clusters, were differentially regulated by BMAA. Protein CcmK (*all0868*) was upregulated, while CcmK (*alr0318*) was downregulated (Table 2). Such different regulation of the same protein, encoded by different genes, may have some adaptive significance and could be influenced by environmental factors.

Obviously, regulation of CO_2_ fixation in nitrogen-replete *Nostoc* was perturbed by BMAA. Moreover, BMAA acted differently on proteins that are involved in the process of CO_2_ fixation in nitrogen-starved cells [23] and in nitrogen-replete cells of *Nostoc* (Table 2 and Table 3). As we have shown earlier [23], two proteins (RbcL (*alr1524*) and CcmM (*all0865*)) involved in CO_2_ fixation were downregulated by BMAA in nitrogen-starved *Nostoc*. However, in the present study, we found that RbcL (*alr1524*), CcmK (*all0868*) and some other proteins were upregulated by BMAA in nitrogen-replete cells. Such a BMAA effect could be explained by PII protein involvement in carbon fixation regulation [28] and by the oppositely directed BMAA regulation of PII in different growth conditions. Thus, this cyanotoxin affects not only nitrogen metabolism, but also leads to changes in carbon metabolism as well.

### 2.4. Starch and Sucrose Metabolism

Photosynthetic carbon is assimilated by cyanobacteria cells via the Calvin cycle in the form of homopolymers of D-glucose, such as glycogen or starch [39,40], which both consist of glucan chains made of glucose residues. Two enzymes involved in starch and sucrose metabolism—4-alpha-glucanotransferase [EC:2.4.1.25] (*alr3871*) and fructokinase [EC:2.7.1.4] (*alr0517*)—were 1.7 times more upregulated in the presence of BMAA in *Nostoc* cells that were grown in nitrate-containing BG11 medium (Table 2, Supplementary Figure S5).

The first enzyme 4-alpha-glucanotransferase belongs to the family of glycosyltransferases and catalyzes a chemical reaction that transfers a segment of a 1,4-alpha-D-glucan to a new position in an acceptor carbohydrate, that could either be glucose or a 1,4-alpha-D-glucan. This enzyme participates in starch and sucrose metabolism in plants [41]. The second enzyme, fructokinase, catalyzes the phosphorylation of D-fructose to D-fructose 6-phosphate (F6P), which is the central and regulatory process in bacteria and plants. This reaction directs fructose to a metabolically active state for glycolysis.

Research on metabolic control in cyanobacteria showed that glycogen and sucrose metabolisms are interconnected in nitrogen-fixing filaments [42,43,44]. Sucrose is known to be a stress-response molecule in cyanobacteria cells. Accumulation of sucrose induced by salt stress was reported [42]. Moreover, an additional role was proposed for sucrose as a carbon carrier molecule, which transports carbon from the vegetative cells to the heterocysts in filamentous nitrogen-fixing strains [42]. In addition, the coordination of sucrose metabolism with nitrogen assimilation at the transcriptional level is supported by experimental evidence, indicating that NtcA also regulates sucrose metabolism genes in *Nostoc* sp. PCC 7120 [43]. Nitrogen fixation is spatially compatible with sucrose synthesis, thereby this disaccharide could be an intermediate in the carbon flow in heterocyst-forming cyanobacteria [44].

Thus, upregulation of these two enzymes may be associated with BMAA upregulation of PII protein and related regulatory proteins in nitrogen-replete cells of *Nostoc* (Figure 1).

### 2.5. Photosynthesis

Cyanobacteria use oxygenic photosynthesis for converting carbon dioxide into different organic molecules [45]. Cyanobacteria are able to regulate their intracellular carbon/nitrogen balance by altering the amount of different photosynthetic proteins. Under different stress conditions, cyanobacteria are able to adapt their photosynthetic apparatus by changing the number of antenna proteins and proteins, which build the structural units of protein complexes of both photosystems. For example, low levels of cytochrome c553 correlate with the decrease in photosynthetic electron transport and with chlorosis [46]. In this proteomic study, it was found that photosynthetic proteins were only slightly affected by BMAA in nitrogen-replete cells of *Nostoc* (Table 2) compared with its very strong action on photosystem PSI proteins in nitrogen-starved cyanobacteria cells [23]. In the present study, five proteins involved in photosynthesis were downregulated and two proteins were slightly upregulated in the presence of BMAA (Table 1 and Table 2). Among the downregulated proteins, we found cytochrome c-550 (*psbV*, *all0259*), subunit IV of photosystem I (*psaE*, *asr4319*), photosystem II protein CP47 (*psbB*, *all0138*), phycobilisome core component apcF (*all2327*) and the beta subunit of ATP synthase F0F1 (*all5039*) (Table 2). Two proteins (cpcB and cpcG4) were slightly upregulated. 

Hence, it can be concluded that after 48 h of BMAA treatment, *Nostoc* cells growing in nitrogen-replete medium started “to feel hungry”. However, this physiological state of cyanobacteria cells cannot be characterized as chlorosis compared with the strong chlorosis they suffer under BMAA treatment during nitrogen starvation conditions [23].

### 2.6. Amino Acid Metabolism

Considering the significant changes caused by BMAA in the number of proteins involved in nitrogen metabolism, bicarbonate transport, CO_2_ fixation and photosynthesis, it could be expected that the changes will also affect the synthesis and metabolism of amino acids. Indeed, proteomic analysis revealed, in nitrogen-replete *Nostoc* cells, multiple changes in the regulation of 10 enzymes, which participate in amino acid metabolism and in amino acid synthesis. Five proteins were downregulated, and five proteins were upregulated (Table 2).

BMAA addition to nitrogen-replete *Nostoc* cells induced changes in the amount of enzymes that are involved in valine, leucine and isoleucine biosynthesis and arginine biosynthesis (Table 2). Two enzymes —valine-pyruvate aminotransferase (EC:2.6.1.66, *alr2811*) and ilvG, acetolactate synthase I/II/III large subunit (EC:2.2.1.6, *all4613*), that participate in valine, leucine and isoleucine biosynthesis—were downregulated (Table 2, Supplementary Figure S6). Note that these two enzymes are under NtcA transcriptional regulation (Table 2).

Two enzymes involved in arginine biosynthesis (argininosuccinate synthase and ornithine carbamoyl-transferase) were strongly downregulated and one enzyme (subunit alpha of urease) was slightly upregulated (Table 2, Supplementary Figure S7). The ornithine carbamoyl-transferase (EC:2.1.3.3, *alr4907)* was absent in *Nostoc* proteome under BMAA treatment (Table 2, Supplementary Figure S7). It is known that a cyanobacteria mutant (*argF*) impaired in this enzyme cannot produce citrulline from arginine [47]. The next enzyme, argininosuccinate synthase (EC:6.3.4.5, *alr4798*), was downregulated (Table 2, Supplementary Figure S7). At the same time, urease [EC:3.5.1.5, *alr3670*] was upregulated.

**Figure 4:**
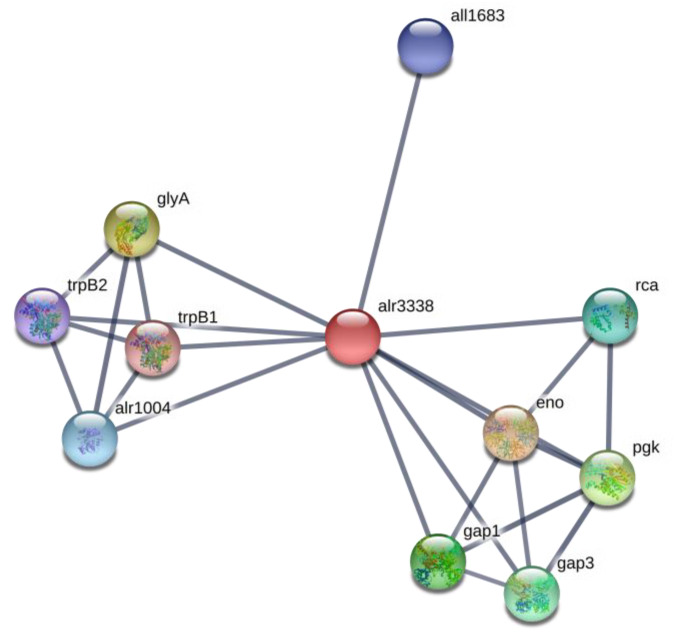
Protein network of phosphoglycerate mutase (alr3338) (red ball) and its protein partners, according to STRING (https://string-db.org). In this figure: eno is enolase that catalyzes the reversible conversion of 2-phosphoglycerate into phosphoenolpyruvate, glyA is a serine hydroxymethyltransferase, which catalyzes the reversible interconversion of serine and glycine, with tetrahydrofolate (THF) serving as the one-carbon carrier, pgk is a phosphoglycerate kinase, it is a glycolytic enzyme, gap1 is a glyceraldehyde-3-phosphate dehydrogenase 1, an enzyme of glycolysis, gap3 is glyceraldehyde-3-phosphate dehydrogenase 3, rca is a ribulose bisphosphate carboxylase/oxygenase activase, alr1004 is alanine-glyoxylate transaminase/serine-glyoxylate transaminase, *all1683* is a phosphoserine aminotransferase, trpB1 is a tryptophan synthase beta chain 1 and trpB2 is a tryptophan synthase beta chain 2.

The enzyme phosphoglycerate mutase/phosphoserine phosphatase (PSP) (EC:3.1.3.3, *alr3338*) was absent (Table 2) in BMAA-treated samples. This enzyme catalyzes one of the terminal steps of the glycolytic pathway—the interconversion of 2-phosphoglycerate and 3-phosphoglycerate—and participates in glycine, serine and threonine metabolism (Supplementary Figure S8). PSP is a key enzyme in the biosynthetic pathway of L-serine [48]. The enzyme catalyzes the dephosphorylation of phosphoserine to serine and inorganic phosphate. The amino acid L-serine is one of the key intermediates of central carbon/nitrogen metabolism. L-serine is involved in the biosynthesis of pyrimidines and purines, and it is an important precursor for other essential cell compounds, including the amino acids glycine and cysteine or phospholipids [49]. It was shown that serine also acts as a metabolic signal that regulates the transcription of photorespiratory genes in plants [50]. The phosphoglycerate mutase (*alr3338*) is involved in protein interactions with other glycolytic enzymes, such as gap1, enolase, and with proteins involved in carbon fixation, such as Rca, Gap3 proteins and with tryptophan synthase, serine hydroxymethyltransferase (GlyA), phosphoserine aminotransferase (*all1683*) and serine-glyoxylate transaminase (*alr1004*) enzymes (Figure 4). Therefore, the absence of phosphoglycerate mutase (*alr3338*) in BMAA-treated cyanobacteria cells may lead to very serious changes in the central carbon metabolism, sulfur metabolism and amino acid metabolism (Supplementary Figure S8).

Furthermore, BMAA perturbed several other amino acid metabolic pathways. Specifically, BMAA disturbed alanine, aspartate and glutamate metabolism pathways as it was also detected in human neuroblastoma cells by metabolic profiling [51]. Two enzymes, glucosamine-fructose-6-phosphate aminotransferase (NodM, *alr3464*) and 1-pyrroline-5 carboxylate dehydrogenase (*alr0540*), were upregulated in the presence of BMAA in nitrogen-replete cells of *Nostoc* (Table 2, Supplementary Figure S9). These two enzymes are involved in glutamate metabolism. The enzyme NodM (*alr3464*) functioning in glutamate metabolism and amino sugars metabolism was upregulated in *Nostoc* under BMAA treatment in all three different growth conditions. So, this enzyme (EC:2.6.1.16) was upregulated after BMAA treatment during nitrogen starvation [23], it was upregulated in nitrogen-replete medium (Table 2) and the protein was upregulated in diazotrophically grown *Nostoc* that already possessed mature heterocysts before adding BMAA [52]. The glutamine-fructose-6-phosphate transaminase catalyzes a chemical reaction, in which the two substrates of this enzyme are L-glutamine and D-fructose 6-phosphate, and the two products are L-glutamate and D-glucosamine 6-phosphate, respectively. Protein NodM participates in the GlnA and GlnB (PII) protein network (Supplementary Figure S10). In our study, we found that the one more protein partner of NodM, the fructokinase [EC:2.7.1.4] (*alr0517*), was also upregulated under BMAA treatment (Table 2). The enzyme 1-pyrroline-5 carboxylate dehydrogenase (EC:1.5.5.21.2.1.88) was upregulated by two-fold (Table 2). This enzyme participates in glutamate metabolism and in arginine and proline metabolism. It is the second enzyme in the proline catabolic pathway [53]. The three substrates of this enzyme are (S)-1-pyrroline-5-carboxylate, NAD^+^, and H_2_O, whereas its three products are glutamate, NADH, and H^+^. In the KEGG (Kyoto Encyclopedia of Genes and Genomes) Database, this enzyme is also described as an RHH (ribbon-helix-helix motif)-type transcriptional regulator and a proline utilization regulon repressor. 

Thereby, BMAA addition upregulated these two enzymes involved in glutamate metabolism. The details of this regulation are still unknown but considering that BMAA and its carbamate are glutamate receptor agonists [54,55,56] and glutamate abolishes BMAA effects on cyanobacteria cells [21,22], it could be suggested that BMAA may mimic glutamate and in some way influence the enzymes participating in glutamate metabolism. The detailed mechanism of such regulation is a subject for future investigations.

Summarizing, it could be concluded that BMAA presence in nitrogen-replete cells of *Nostoc* leads to remarkable changes in amino acid metabolic pathway regulation, including those where glutamate and 2-oxoglutarate participate.

### 2.7. Signaling and Stress Response Proteins, Proteases and Chaperones

Significant disturbance of the main metabolic pathways caused by BMAA led to upregulation of several signaling and stress response proteins, various proteases and chaperones in nitrogen-replete cells of *Nostoc* (Table 2). Here, several proteins were downregulated or were present only in the control samples.

ClpP protease (*alr3683*) and serine proteinase (*alr2758*) were upregulated almost two-fold. It is known that proteolysis is an essential cellular activity that mediates protein turnover and the degradation of undesired proteins in the intracellular environment. It is well known that ClpP protease is involved in the proteolysis of defective and misfolded proteins [57]. Upregulation of Clp may indicate that the number of targets of this protease also increased in the presence of BMAA. In addition to ClpP protease, another enzyme, serine proteinase (*alr2758*), was upregulated as well. Serine proteases are common proteases and they cleave primarily at Gln–Gly, Gln–Ser, Gln–Ala, and Gln–Asn bonds [58]. As we reported previously, the effect of BMAA on eukaryotic and prokaryotic cells is pleiotropic and can involve different mechanisms [15]. One such mechanism, for example, is the disturbance of protein synthesis. It was shown that BMAA can be mistakenly incorporated into proteins instead of L-serine in human cells [59]. Recently, it was shown that BMAA can be a substrate for human alanyl-tRNAsynthetase (AlaRS) and can form BMAA-tRNAAla by escaping from the intrinsic AlaRS proofreading [60]. Additionally, it was found by using the AlaRS from *Nostoc* sp. PCC 7120 that cyanobacteria AlaRS also activates BMAA [60]. It can therefore be assumed that a possible increase in the amount of defective and misfolded proteins in the presence of BMAA may be the reason for the upregulation of serine protease.

Another metabolically important enzyme, upregulated in the presence of BMAA, was identified as Cytochrome P450 (*all1361*) (Table 2). It is known that Cytochrome P450 monooxygenases (CYPs/P450s) is a diverse superfamily of heme-dependent enzymes that contribute to the production and diversity of various secondary metabolites and are present in all cyanobacteria [61]. These enzymes introduce oxygen into a wide range of molecules. The bacterial CYPs/P450s can perform many biochemical reactions: alkene epoxidation, aliphatic hydroxylation, oxidative phenolic coupling, aromatic hydroxylation, heteroatom oxidation and dealkylation, and multiple oxidations including C-C bond cleavage [62]. Investigation of its function(s) in cyanobacteria cells is a subject for future studies.

The aldo/keto reductase (*all2316*) was two-fold upregulated in the presence of BMAA (Table 2). This enzyme reduces carbonyl substrates such as: sugar aldehydes, keto-steroids, keto-prostaglandins, retinals, quinones and lipid peroxidation by-products. Aldo/keto reductases are pluripotent enzymes that function in the metabolism of endogenous substrates and xenobiotics [63].

Two enzymes, involved in glutathione metabolism, were also upregulated by treatment with BMAA in cyanobacteria cells. They are glutathione reductase (NADPH) [EC:1.8.1.7] and leucyl aminopeptidase [EC:3.4.11.1]. Glutathione (GSH) is an antioxidant which prevents damage of cellular components by reactive oxygen species and participates in thiol protection and redox regulation of cellular thiol proteins under oxidative stress by protein S-glutathionylation [64]. Glutathione is also involved in the detoxification of formaldehyde, a toxic metabolite produced under oxidative stress and also under BMAA decay. It was shown that the reaction of BMAA with pyridoxal-5′-phosphate produced methylamine and ammonia as final products. Then, the methylamine is oxidized to formaldehyde, hydrogen peroxide and ammonia [65,66]. Therefore, activization of glutathione-related enzymes can be explained by metabolic disorder, induced by BMAA, as well as BMAA decomposition in cyanobacteria cells.

Recombinase A, RecA (*all3272*), was upregulated almost four-fold under BMAA treatment in *Nostoc* cells (Table 2). The same upregulation effect of BMAA on this protein was found in our previous proteomic study [23]. It was suggested that oxidative stress, induced by BMAA, may lead to DNA damage and apoptosis and therefore may induce DNA repair cell activity and SOS response. Cyanobacteria have many *Escherichia coli*-like genes, that participate in DNA recombination and repair [67], but they are much less studied than genes for the *E. coli* reparation system.

**Figure 5:**
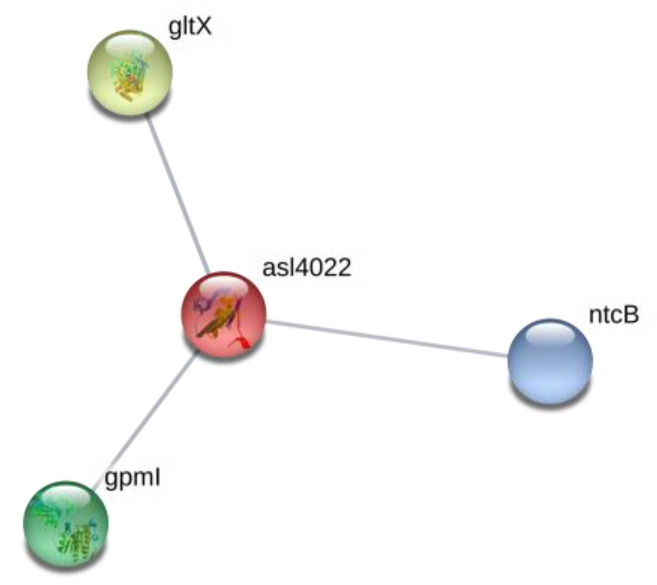
Protein network of rbpD, a RNA-binding protein (*asl4022*), and its protein partners (https://string-db.org). In this figure: protein gltX is Glutamate-tRNA ligase, which catalyzes the attachment of glutamate to tRNA(Glu) in a two-step reaction—glutamate is at first activated by ATP to form Glu-AMP and then is transferred to the acceptor end of tRNA(Glu). Note that gltX belongs to the class-I aminoacyl-tRNAsynthetase family and to Glutamate-tRNA ligase type 1 subfamily. NtcB is the nitrite-responsive transcriptional enhancer, and gpmI is 2,3-bisphosphoglycerate-independent phosphoglycerate mutase that catalyzes the interconversion of 2-phosphoglycerate and 3-phosphoglycerate.

Two RNA-binding proteins (rbpD, *asl4022* and mRNA-binding protein, *alr4831*) were identified as upregulated proteins in nitrogen-replete *Nostoc* cells under BMAA action (Table 2). One of them, rbpD, RNA-binding protein (*asl4022*), was upregulated almost three-fold. It should be noted that protein rbpD has three protein partners (Figure 5), where two of them, NtcB and gltX, are involved in nitrogen metabolism of cyanobacteria. NtcB is a LysR-family protein and is required in addition to NtcA, a CAP-family (catabolite activator protein) protein, for the expression of genes, which encode proteins specifically involved in nitrate assimilation in *Nostoc* sp. PCC 7120 [68]. The other protein, glutamyl-tRNA synthetase (gltX, *all3205*), is involved in protein and pyrrole derivative biosynthesis. Moreover, in the case of cyanobacteria *Synechococcus* sp. PCC 7942, it was shown that transcription of the gltX gene was activated in the presence of ammonium or nitrate but not in nitrogen-free medium [69]. NtcA is required for full gltX expression, but it is not required for basal constitutive transcription of this gene, which is consistent with the essential role of gltX in protein and pyrrole derivative biosynthesis. It was shown that gltX— a protein involved in the incorporation of glutamate into protein synthesis—is under NtcA control. Thus, the transcriptional factor NtcA orchestrates coordinated regulation of every essential aspect of nitrogen metabolism [70]. In our previous proteomic study [23], we found that gltX was absent in nitrogen-starved *Nostoc* cells under BMAA treatment.

Two more proteins, uncharacterized conserved protein YggE (*all0089*) and MBL (Metallo-Beta-Lactamase domain) fold metallo-hydrolase (*all0268*), have been identified only in the control samples of *Nostoc* (Table 2). They were absent in the BMAA-treated samples. Protein YggE is poorly characterized and there is no information about this protein so far. We found only that YggE is under NtcA transcriptional regulation (Table 2). The other protein, MBL fold metallo-hydrolase, has also been scantly characterized. It is known that it binds to trpG protein (*all0269*) (https://string-db.org). This protein partner of YggE, anthranilate synthase, belongs to a large group of biosynthetic enzymes that are able to catalyze the removal of the ammonia group from glutamine and the further transfer of this group to a substrate to form a new carbon-nitrogen group. This catalytic activity is known as glutamine amidotransferase (GATase) [71]. Hence, we can suggest that the absence of MBL fold metallo-hydrolase (*all0268*) in BMAA-treated *Nostoc* samples can have an impact on its protein partner, trpG protein, which is involved in glutamine-glutamate metabolism.

Thioredoxin I was also absent in BMAA-treated *Nostoc* cells (Table 2). The thioredoxin I is a small disulfide-containing redox protein that reduces disulfide bonds in other proteins. Unlike other bacteria, cyanobacteria have two distinct thioredoxins [72]. Thioredoxin I is similar to bacterial thioredoxins, while thioredoxin II is specific for cyanobacteria. Different enzymes, involved in CO_2_ fixation, carbon catabolism and nitrogen metabolism, are regulated by a disulfide redox mechanism that can be affected by thioredoxins [72,73]. Among them, there is glutamine synthetase (GS) [72].

Note that, as it was found in our previous proteomic study [23], thioredoxin reductase (EC:1.8.1.9) upregulates under BMAA treatment in nitrogen-starved *Nostoc* cells (Table 3). Therefore, we can see that BMAA impact is different on the thioredoxin enzymes in cyanobacteria *Nostoc* in the presence and in the absence of nitrogen in the growth medium.

### 2.8. Hypothetical Proteins

In our proteomic study, we have found 18 hypothetical proteins (Table 1 and Supplementary Table S2). The identification of hypothetical proteins in proteomic studies is usually somewhat rather disappointing for researchers. Everyone wants to know the exact protein functions. However, it is possible to find some useful information about certain hypothetical proteins by using the ALCOdbCyano database (http://alcodb.jp/cyano/), where co-expressed gene lists are presented (Supplementary Table S3). Information about co-expressed genes may provide a key to understanding the possible function of hypothetical protein. Moreover, we have found that several identified hypothetical proteins were in the same list with other proteins also identified in the present proteomic study (Supplementary Table S3, marked green). Note also that several hypothetical proteins were identified both in this study and in our previous proteomic study [23] (Table 3).

Among the upregulated hypothetical proteins, were identified proteins whose genes are co-expressed with the genes that encode chaperones, regulatory proteins, endopeptidases, DNA-binding proteins and others (Supplementary Table S3). For example, the gene encoding a hypothetical protein (*alr4505*) is co-expressed together with the gene *alr2991* (Dna J protein). Note that DnaJ proteins, also called J-domain proteins, function as molecular co-chaperones and play an important role in protein folding [74]. This hypothetical protein may be involved in chaperone activity—it is upregulated 3.5 times (Supplementary Table S2). This assumption is consistent with the observation that BMAA has a destabilizing effect on protein synthesis in cyanobacteria cells (see Section 2.7) and, therefore, chaperone activity should be required in cells. In addition, gene *alr4505* is under NtcA transcriptional control as well as *all1411* and *asr1156* genes (Supplementary Table S2). Gene *all1411*, which encodes the hypothetical protein, is in the same list with the gene encoding the carbon dioxide-concentrating mechanism protein CcmK. This hypothetical protein is upregulated alongside the CcmK protein that was identified in our study (Table 2). The gene *asr1156*, which encodes an upregulated hypothetical protein, is expressed together with the gene *asr3935* (DNA-binding protein HU, Table 2), gene *alr2818* (heterocyst differentiation protein HetP) and with gene *alr4392* (nitrogen-responsive regulatory protein NtcA). 

Among downregulated hypothetical proteins, there were also several interesting candidates for further analysis. They are hypothetical proteins, encoded by genes *all1338*, *asl4369* and *all4580*. These proteins were identified only in the control samples and not in the BMAA-treated samples (Supplementary Table S2). All these and other hypothetical proteins can be investigated in upcoming studies by using insertional mutagenesis and transcriptional analysis.

## 3. Conclusions

**Figure 6:**
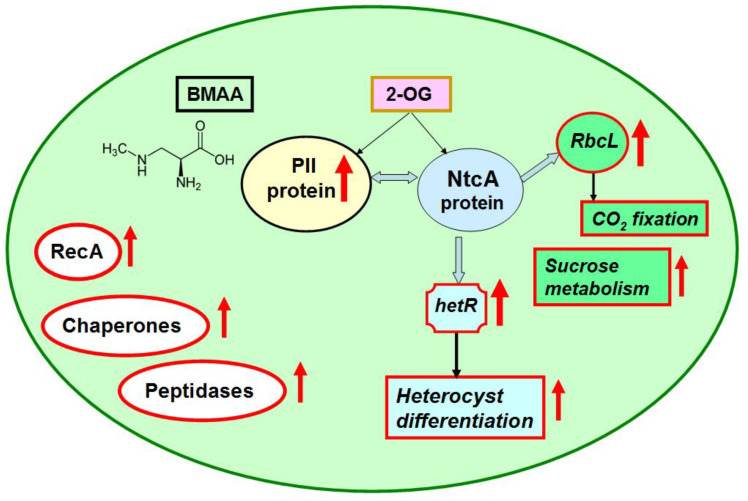
The main targets of BMAA action in nitrogen-replete *Nostoc* cell are presented. Red arrows stand for upregulation of the main proteins (written in circles), gene *hetR* (written in a curved square) and altered cellular processes (written in red rectangles) that were identified in this study and in our previous work [22].

This work continues our previous studies on the molecular mechanisms of BMAA’s effect on cyanobacteria cells under different growth conditions. We discovered the pleiotropic regulatory effect of BMAA on *Nostoc* sp. PCC 7120 proteome under nitrogen-replete conditions to be quite different from what we had found previously in nitrogen-starved growth conditions [23] (Table 3). In cyanobacteria cells, the most significant difference in proteome expression between the BMAA-treated and untreated samples under different growth conditions was detected in key regulatory protein PII. BMAA downregulated PII protein in nitrogen-starving conditions and upregulated PII in nitrogen-replete conditions. This could be the main reason behind a specific regulatory effect of BMAA on heterocyst formation and heterocyst- and nitrogenase-related gene expression that we have discovered in *Nostoc* by using RT-PCR and microscopy analysis in our previous studies [21,22]. It is known that changes in the state and quantity of PII protein lead to changes in the regulation of many other proteins, including different enzymes and transcriptional factors [28]. Moreover, 15 differently expressed proteins, encoded by genes, which are under the transcriptional control of the global regulator NtcA, were identified in this work. Note that NtcA is one of the main protein partners and transcriptional regulators of PII protein. Thereby, the presented results indicate that the main primary targets for the BMAA effect appear to be metabolic processes involving 2-oxyglutarate, glutamate and regulatory proteins PII and NtcA (Figure 6). Complex changes were also noticed in other key regulatory and metabolic proteins (RbcL, RbcS, Rca, cmpA, gltS, NodM, thioredoxin 1, RpbD, ClpP, MinD, RecA, etc.). We have demonstrated that in *Nostoc* cells, under nitrogen-replete conditions, BMAA effects proteins involved in photosynthesis, carbon fixation and carbon dioxide-concentrating mechanisms, amino acids metabolism (including enzymes participating in glutamate-glutamine turnover), protein synthesis, in starch and sucrose metabolism and in fatty acids synthesis. This severe metabolic imbalance could be responsible for the “starvation” state in nitrogen-replete cyanobacteria cells in the presence of BMAA, which could possibly be the cause of unexpected heterocyst-like cell formation [22].

Moreover, this metabolic imbalance leads to intracellular stress response. This stress response was confirmed by the upregulation of stress defense proteins, including a DNA repair enzyme, RecA. Almost a four-fold increase in the amount of RecA indicates activation of the “SOS” response mechanism in cyanobacteria cells. Similar stress was also detected in nitrogen-limited conditions [23]. Therefore, BMAA induces intracellular stress in both nitrogen-limited and in the nitrogen-replete conditions.

These results provide novel insight into the regulation of cyanobacterial metabolism affected by BMAA and highlight new opportunities for upcoming experimental studies. 

## 4. Materials and Methods

### 4.1. Cyanobacterial Strain and Cultivation Conditions

Cyanobacterium *Nostoc* sp. PCC 7120 was received from the Pasteur Culture collection of Cyanobacteria, Paris, France. Cyanobacterium was grown at a light intensity of 18 µmol photons m^−2^ s^−1^ on a shaker with continuous shaking at 63 rpm and at 25 °C. *Nostoc* was grown in 100 mL Erlenmeyer flasks containing 25 mL of sodium nitrate containing BG11N medium [75] for 3 days. Then, cells were washed 3 times with BG11N [75], and afterwards, cyanobacterium was grown in BG11N medium for 48 h in two experiments: (1) the control samples were grown without addition of water solution of beta-N-methylamino-L-alanine (L-BMAA) (Cat no. B-107, Sigma-Aldrich, Saint Louis, MO, USA) and (2) the treated samples were grown with the addition of water solution of BMAA (20 µM), as performed earlier [22]. This concentration is in the range of environmental concentrations of BMAA [6,10,15]. Cells were collected by centrifugation at 5000 rpm and 4 °C for 10 min and frozen at −80 °C until being used for proteomic analysis. Experiments were performed in three independent biological replicates.

Time of cell treatment/incubation with and without BMAA (48 h) was selected according to our previously published studies [22,23].

### 4.2. Trypsin Digestion in Solution

Cellular pellet was treated with lysozyme (0.3 mg/mL) (Sigma) for 60 min at 4 °C and resuspended in 100 uL 100 mMtris-HCl buffer, pH 8.0, with the addition of Protease inhibitor Mix (GE Healthcare), 0.1% sodium deoxycholate (DCNa) (Sigma) and 2.5 mM EDTA (Sigma). Cells were lysed by six cycles of 30 s sonication (Cell Disruptor, Branson Ultrasonics Corp, Danbury, CT, USA) and 5 min incubation at 4 °C. After that, dry urea and DCNa were added to the sample to final concentrations of 8 M and 1%, respectively. After incubation for 20 min, the sample was centrifuged at 14,000 rpm for 10 min at 4 °C to remove intact cells. The supernatant was selected, and protein concentration was estimated using a Bradford Protein Assay Kit (BioRad, Hercules, CA, USA). Protein cysteine bonds were reduced in the supernatant by the addition of 5 mM Tris (2-carboxyethyl) phosphine hydrochloride (TCEP) (Sigma) for 60 min at 37 °C and, subsequently, alkylated with 30 mM iodoacetamide (BioRad, Hercules, CA, USA) at room temperature in the dark for 30 min. The step in which TCEP was added was repeated. Then, the sample was diluted 6-fold with 50 mM Tris-HCl, pH 8.0, with 0.01% DCNa. Trypsin (Trypsin Gold, Mass Spectrometry Grade, Promega, Madison, WI, USA) was added in 1/50 w/w trypsin/protein ratio and incubated at 37 °C overnight. To stop trypsinolysis and degrade the acid-labile DCNa, trifluoroacetic acid (TFA) (Sigma, Saint Lois, MO, USA) was added to a final concentration of 0.5% *v*/*v* (the pH should be less than 2.0), incubated at 37 °C for 45 min, and the samples were centrifugated at 14,000 × *g* for 10 min to remove the DCNa. Peptide extract was desalted using a Discovery DSC-18 Tube (Supelco, Merck KGaA, Darmstadt, Germany) according to the manufacturer’s protocol. Peptides were eluted with 1 mL 75% acetonitrile (Sigma) with 0.1% TFA, dried in a SpeedVac (Labconco, Kansas City, MO, USA) and resuspended in 3% acetonitrile with 0.1% TFA to a final concentration of 5 μg/μL.

### 4.3. Liquid Chromatography with Tadem Mass Spetrometry (LC-MS/MS) Analysis 

The analysis was performed on a Triple Time-of-Flight (TOF) 5600+ mass spectrometer with a NanoSpray III ion source (AB Sciex, Framingham, MA, USA) coupled with a NanoLC Ultra 2D+ nano-high performance liquid chromatography (HPLC) system (Eksigent, now part of Sciex, Framingham, MA, USA), as we have described previously [23,76]. The HPLC system was set in trap-elute mode. The sample loading buffer and the buffer A were made from a mixture of 98.9% water, 1% methanol and 0.1% formic acid (*v*/*v*). Buffer B contained 99.9% acetonitrile and 0.1% formic acid (*v*/*v*). Samples were injected in a Chrom XP C18 trap column (3.6μm, 120 Å, 350 μm × 0.5 mm; Eksigent, Dublin, CA, USA) at a flow rate of 3 μL/min for 10 min and afterwards eluted through a 3C18-CL-120 separation column (3 μm, 120 Å, 75 μm × 150 mm; Eksigent) at a flow rate of 300 nL/min. We started with a gradient from 5% buffer B (0 min) to 40% buffer B (90 min), followed by a gradient change to 95% buffer B for 10 min and a re-equilibration with 5% buffer B during 20 min. Two blank runs over 45 min consisting of 5 - 8 min cycles (5% B, 95%, 95%, 5%) were performed between the different samples in order to wash the system and to prevent carryover. 

The information-dependent mass-spectrometer analysis included one survey scan of parent ions (MS1) that was followed by 50 dependent scans of fragment ions (MS2). MS1 acquisition parameters were set as follows: the mass range was 300–1250 *m*/*z*, and the signal accumulation time was 250 ms. Ions for MS2 analysis were chosen on the basis of intensity with a charge state from 2 to 5 and a threshold of 200 counts per second. MS2 acquisition parameters were set as follows: the resolution of the quadrupole was UNIT (0.7 Da), the measurement mass range was 200–1800 *m*/*z* and the signal accumulation time was 50 ms for each parent ion. Collision-activated dissociation was carried out with nitrogen gas and the collision energy ranged was set up from 25 to 55 V within the signal accumulation time of 50 ms. Analyzed parent ions were sent to the dynamic exclusion list for 15 s in order to obtain an MS2 spectra at the chromatographic peak apex. β-Galactosidase tryptic solution (20 fmol) was run with gradient (5% to 25% buffer B) for 15 min between every two samples and between sample sets in order to calibrate the mass spectrometer and to control the overall system performance, stability and reproducibility.

### 4.4. Protein Identification by LC-MS/MS Data Analysis

For protein identification and semi-quantitative spectral counting, all LC-MS/MS data were searched against the National Center for Biotechnology Information (NCBI) GenBank protein sequence database for *Nostoc* sp. PCC 7120 also containing common contaminant proteins. Identification of proteins was performed with ProteinPilot (version 4.5, SciexABSciex, Forster, CA, USA) in an identification mode with the following parameters: Cys alkylation by iodoacetamide, trypsin digestion, TripleTOF 5600 instrument, false discovery rate (FDR) analysis, and thorough an ID search with a detected protein threshold of 95.0%. Protein identification was considered significant if the estimated local false discovery rate was 1% or lower, and at least 2 different peptides were identified for the protein with a confidence score above 95%. Spectral counting was performed with in-house script under emPAI [77] protocol with only tryptic peptides with local FDR ≤ 1% taken into account. 

Quantitative analysis was performed with MaxQuant against the same database. The settings used were as follows: a standard label-free analysis, fixed cysteine carbamidomethylation (which is allowed for use in quantitation), no variable modifications, default settings for Sciex Q-TOF instrument for MS and MS/MS spectra processing, tryptic digest with cleavage allowed only after lysine or arginine residues, unless they are followed by proline residue and with 0 missed sites allowed, label-free quantification with minimum 2 label-free quantification (LFQ) ratios, normalization performed and missing peaks re-quantified, minimum peptide length 7, maximum peptide mass 4600 Da, only unique peptides used for quantification. The PSM and protein FDR threshold was set to 5%, and at least 1 unique peptide was required for the protein group. Statistical significance of observed differences in each case was assessed with Welch’s 2-sided *t*-test with Benjamini–Yekutieli adjustment for multiple comparisons, with *p*-value thresholds of 0.05 and 0.1.

### 4.5. Pathway Analysis Based on LC-MS/MS Data

The significantly altered proteins obtained from LC-MS/MS data analysis were subjected to analysis using the UniProt Knowledgebase (https://web.expasy.org/docs/userman.html#what_is_sprot) and the Kyoto Encyclopedia of Genes and Genomes (KEGG) pathways database (https://www.genome.jp/kegg/pathway.html). 

Protein–protein interactions were analyzed by STRING (Protein–Protein Interaction Networks Functional Enrichment Analysis; https://stringdb.org/cgi/download.pl?sessionId=LdNVdFoNwm9Q). Gene co-expression data for *Nostoc* sp. PCC 7120 were obtained from ALCOdbCyano (http://alcodb.jp/cyano/). The co-expression data in this database were calculated using 116 microarray data items downloaded from the KEGG EXPRESSION Database (https://www.genome.jp/kegg/expression/). Sequence information and gene annotations were retrieved from CyanoBase (http://genome.microbedb.jp/mnt.html).

NtcA-regulated genes were found with the CollecTF database (a database of transcription factor binding sites (TFBS) in the Bacteria domain) (http://www.collectf.org/browse/home/) [78].

## Figures and Tables

**Table 1:** The results of β-N-methylamino-L-alanine (BMAA) effect on the protein profile of *Nostoc* during growth in a nitrogen-replete medium. The number of upregulated and downregulated proteins is quantified according to label-free quantification (LFQ) ratio of BMAA-treated sample/Control sample.

No.	Pathway	Number of Proteins Affected by BMAA	Total Amount	
			Upregulated	Downregulated
1	Nitrogen metabolism	2	1	1
2	Photosynthesis	7	2	5
3	CO_2_ fixation and CO_2_-concentrating mechanism	7	3	4
4	Cell division	2	0	2
5	Regulatory proteins, proteases	10	5	4
6	Transporters	2	2	0
7	Stress response	6	5	1
8	Translation	7	5	2
9	Amino acid synthesis and metabolism	10	5	5
10	Purine and Pyrimidine metabolism	4	2	2
11	Starch and sucrose metabolism	2	2	0
12	Sulfur metabolism	1	0	1
13	Fatty acid biosynthesis	1	1	0
14	Energy metabolism	2	1	1
15	Hypothetical proteins	18	10	8
	Total	80	44	36

**Table 2:** BMAA effect on the protein profile of *Nostoc* during growth in nitrogen-replete medium. The fold changes between the BMAA-treated samples and the control samples are shown, (*p* < 0.1). Statistical significance of the observed differences in each case was calculated by the Welch’s 2-sided *t*-test with the Benjamini–Yekutieli adjustment for multiple comparisons with *p*-value thresholds of less than 0.1. (*) Genes that are under transcriptional control of the global nitrogen regulator NtcA (according to the CollecTF database).

No.	Protein	Gene	Function	Fold Change LFQ Ratio BMAA-Treated/Control		*p*-Value
**Nitrogen Metabolism (Two Proteins)**						
1	GlnB|P-II	all2319*	Nitrogen regulatory protein	1.75		0.0999
2	glutamate synthase (ferredoxin) [EC:1.4.7.1]|(GenBank) gltS; ferredoxin-glutamate synthase	*alr4344*	Nitrogen metabolism and Glyoxylate and dicarboxylate metabolism		0.71	0.0226
**Photosynthesis (7 Proteins)**						
3	cpcB	*alr0528*	phycocyanin beta chain	1.14		0.0909
4	cpcG4	*alr0537*	phycobilisome rod-core linker protein	1.18		0.0625
5	EC:7.1.2.2,7.2.2.1 atpB ATP synthase F0F1 subunit beta F-type H^+^/Na^+^-transporting ATPase subunit beta	*all5039*	Oxidative phosphorylation, Photosynthesis		0.83	0.0858
6	psbB	*all0138*	photosystem II CP47 protein		0.82	0.0717
7	psbV	*all0259*	cytochrome c-550		0.71	0.0533
8	apcF	*all2327*	phycobilisome core component		0.82	0.0533
9	psaE; photosystem I protein E	*asr4319*	photosystem I subunit IV		0.69	0.0040
**CO_2_ Fixation and Carbon Dioxide Concentration Mechanism (7 Proteins)**						
10	RbcL [EC:4.1.1.39]	alr1524*	CO_2_ fixation ribulosebisophosphate carboxylase large chain	1.67		0.0535
11	Rca	alr1533*	CO_2_ fixation RuBisCO activase	1.79		0.0112
12	CcmK	*all0868*	CO_2_ fixation Carbon dioxide-concentrating mechanism protein	1.82		0.0103
13	RbcS [EC:4.1.1.39]	alr1526*	CO_2_ fixation Ribulose bisphosphate carboxylase small chain		0.68	0.0148
14	CcmK	*alr0318*	CO_2_ fixation Carbon dioxide-concentrating mechanism protein		0.76	0.0533
15	CmpA bicarbonate transport bicarbonate-binding protein	*alr2877*	Part of the ABC transporter complex Cmp ABCD involved in bicarbonate transport, binds bicarbonate with high affinity		found only in control sample	0.0228
16	transketolase [EC:2.2.1.1]	*alr3344*	Pentose phosphate pathway Carbon fixation in photosynthetic organisms		0.79	0.0669
**Cell Division (Two Proteins)**						
17	FtsH	*all3642*	cell division protein		0.76	0.0588
18	MinD	*alr3456*	septum site-determining protein		0.67	0.0008
**Regulatory Proteins and Proteases (9 Proteins)**						
19	ClpP EC:3.4.21.92	*alr3683*	ATP-dependent protease ClpP proteolytic subunit	1.85		0.0378
20	Serine proteinase	*alr2758*	modification, protein turnover, chaperones	1.82		0.0062
21	RbpD|RNA-binding protein	*asl4022*		2.7		0.0339
22	mRNA-binding protein	*alr4831*		1.72		0.0630
23	Cytochrome P450	*all1361*	Cytochrome P450 monooxygenases is a heme-dependent enzyme that introduces oxygen into a wide range of molecules	1.49		0.0177
24	YggE, uncharacterized conserved protein	all0089*	It contains kinase-interacting SIMPL domain		found only in control sample	0.0162
25	MBL fold metallo-hydrolase	*all0268*	https://www.ncbi.nlm.nih.gov/Structure/cdd/cl23716		found only in control sample	0.0037
26	RpaA DNA-binding response regulator, OmpR family	all0129*	two-component system, OmpR family, response regulator		0.83	0.0089
27	DNA-binding protein HU, hanA	*asr3935*	histone-like DNA-binding protein HU		0.70	0.0007
**Transporters (Two Proteins)**						
28	ABC transporter ATP-binding protein	*alr2372*	ABC-2-type transport system ATP-binding protein	1.49		0.0408
29	ABC transporter ATP-binding protein	alr1554*	ATP-binding cassette, subfamily B	3.23		0.0375
**Stress Response (** **6 Proteins)**						
31	thioredoxin 1	*all1866*	Chaperones and folding catalysts, reduces disulfide bonds in other proteins		found only in control sample	0.0489
32	RecA	*all3272*	recombinase A	3.7		0.0417
33	Glutathione reductase (NADPH) [EC:1.8.1.7]	*all4968*	Glutathione metabolism	1.49		0.0202
34	leucylaminopeptidase [EC:3.4.11.1]	*alr0237*	Glutathione metabolism	1.49		0.0469
35	aldo/ketoreductase	*all2316*	Positive role of AKR in detoxification of reactive carbonyl species (RCS) produced under oxidative stress	2.04		0.0143
36	polyribonucleotidenucleotidyltransferase [EC:2.7.7.8]	*all4396*	RNA degradation	1.28		0.0632
**Translation (7 Proteins)**						
37	IF-2	*alr3832*	translation initiation factor	1.23		0.0395
38	50S ribosomal protein L16	*all4208*	large subunit ribosomal protein L16	1.54		0.0142
39	RpsP protein	*asr1953*	small subunit ribosomal protein S16	3.33		0.0007
40	DNA-directed RNA polymerase subunit omega [EC:2.7.7.6]	*asr4648*	Promotes RNA polymerase assembly	1.61		0.0073
41	isoleucyl-tRNAsynthetase [EC:6.1.1.5]	*alr1073*	Aminoacyl-tRNA biosynthesis	1.47		0.0313
42	protein S13	all4193*	small subunit ribosomal		0.46	0.0255
43	protein S10	*all4336*	small subunit ribosomal		0.68	0.0201
**Amino Acid Synthesis and Metabolism (10 Proteins)**						
44	valine-pyruvate aminotransferase [EC:2.6.1.66]	alr2811*	Valine, leucine and isoleucine biosynthesis		0.625	0.0887
45	ilvG, acetolactate synthase I/II/III large subunit [EC:2.2.1.6]	all4613*	Valine, leucine and isoleucine biosynthesis		0.51	0.0315
46	argininosuccinate synthase [EC:6.3.4.5]	*alr4798*	Arginine biosynthesis Alanine, aspartate and glutamate metabolism		0.72	0.0704
47	phosphoserine phosphatase [EC:3.1.3.3]	*alr3338*	Glycine, serine and threonine metabolism		found only in control sample	0.0191
48	ornithine carbamoyltransferase [EC:2.1.3.3]	*alr4907*	Arginine biosynthesis		found only in control sample	0.0015
49	NodM, glutamine-fructose-6-phosphatetransaminase (isomerizing) [EC:2.6.1.16]	*alr3464*	Alanine, aspartate and glutamate metabolism	1.43		0.0113
50	Urea subunit alpha [EC:3.5.1.5]	*alr* *3670*	Arginine biosynthesis Purine metabolism	1.59		0.0648
51	murE UDP-N-acetylmuramoyl-L-alanyl-D-glutamate-2,6-diaminopimelateligase [EC:6.3.2.13] |	*all1663*	Lysine biosynthesis Peptidoglycan biosynthesis	1.27		0.0623
52	RHH-type transcriptional regulator, proline utilization regulon repressor/proline dehydrogenase/delta 1-pyrroline-5-carboxylate dehydrogenase [EC:1.5.5.21.2.1.88]	*alr0540*	Alanine, aspartate and glutamate metabolism Arginine and proline metabolism	1.79		0.0362
53	cysteine synthase [EC:2.5.1.47]	all2521*	Cysteine and methionine metabolism Sulfur metabolism	1.72		0.0266
**Nucleotide Synthesis (4 Proteins)**						
54	phosphoribosylformyl- glycinamidine cyclo-ligase [EC:6.3.3.1]	*alr3525*	Purine metabolism		0.67	0.0605
55	Phosphoribosylamine- glycine ligase [EC:6.3.4.13]	*alr3510*	Purine metabolism		found only in control sample	0.0005
56	nucleoside-diphosphate kinase [EC:2.7.4.6]	*alr3402*	Purine metabolism Pyrimidine metabolism	1.92		0.0212
57	uracil phosphoribosyltransferase [EC:2.4.2.9]	*all2063*	Pyrimidine metabolism	2.04		0.0528
**Starch and Sucrose Metabolism (Two Proteins)**						
58	4-alpha-glucanotransferase [EC:2.4.1.25]	*alr3871*	Starch and sucrose metabolism	1.69		0.0008
59	fructokinase [EC:2.7.1.4]	*alr0517*	Fructose and mannose metabolism Starch and sucrose metabolism	1.69		0.0837
**Sulfur Metabolism (One Protein)**						
60	phosphoadenosinephosphosulfate reductase [EC:1.8.4.8 1.8.4.10]	*all4464*	Sulfur metabolism		0.65	0.0151
**Fatty Acid Biosynthesis (One Protein)**						
61	3-oxoacyl-[acyl-carrier protein] reductase [EC:1.1.1.100]	*alr1894*	Fatty acid biosynthesis Biotin metabolism	2.86		0.0316
**Energy Metabolism (Two Proteins)**						
62	NAD(P)H-quinone oxidoreductase subunit J [EC:7.1.1.2]	*all3840*	Oxidative phosphorylation	found only in BMAA-treated sample		0.0940
63	inorganic pyrophosphatase [EC:3.6.1.1]	all3570*	Oxidative phosphorylation		0.77	0.0806

**Table 3:** A comparison of the BMAA action on the sets of different proteins that were identified in nitrogen-starved [23] and nitrogen-replete cells of *Nostoc.*

Protein	The Fold Changes between the BMAA-Treated Samples and Control Sample *			
	Nitrogen Starvation Growth Conditions (Previous Study [23])		Nitrogen-Replete Growth Conditions (Present Study)	
	Downregulation	Upregulation	Downregulation	Upregulation
PII	0.55			1.75
rbcL	0.67			1.67
ccmK				1.82
ccmM	0.64			
Urease subunit alpha	0.82			1.59
thioredoxin I			Control	
thioredoxin reductase		2.22		
S10	0.068		0.68	
RecA		3.03		3.7
nodM		2.2		1.43
Alr4505		6.67		3.57
All1411		4.55		2.7
Alr3297	0.67			1.29
Asl4369	Control		Control	

* The fold changes between the BMAA-treated samples and control sample are shown, (*p* < 0.1). The word “Control” indicates that the protein was present only in the control samples.

## Supplementary Materials

The following supplementary materials are available online at https://www.mdpi.com/2072-6651/12/6/372/s1: Figure S1: The impact of BMAA on nitrogen metabolism in nitrogen-replete cells of *Nostoc* 7120, Figure S2: The impact of BMAA on GltS (*alr4344*) in nitrogen-replete cells of *Nostoc* 7120, Figure S3: The impact of BMAA on rbcL (*alr1524*) and on rbcS (*alr1526*) subunits of Rubisco in nitrogen-replete cells of *Nostoc* 7120, Figure S4: Protein network of ccmK and its protein partners, Figure S5: BMAA upregulates proteins involved in starch and sucrose metabolism of nitrogen-replete cells of *Nostoc* 7120, Figure S6: BMAA downregulates enzymes that are involved in valine, leucine and isoleucine biosynthesis, Figure S7: The impact of BMAA on arginine biosynthesis, Figure S8: The impact of BMAA on glycine, serine and threonine metabolism, Figure S9: BMAA disturbs alanine, aspartate and glutamate metabolism pathways, Figure S10: Protein network of glucosamine-fructose-6-phosphate aminotransferase (nodM, *alr3464*) and its protein partners, according to STRING. Table S1: Original proteomic data, Table S2: Results of BMAA effects on hypothetical protein profile of *Nostoc* sp. PCC 7120 during growth in nitrogen-replete medium, Table S3: Gene co-expression data for identified hypothetical proteins in the proteome of *Nostoc* sp. PCC 7120 under BMAA treatment while growing in nitrogen-replete medium according to ALCOdbCyano.
