# Supplementary material for: Proteomic Insights into Starvation of Nitrogen-Replete Cells of Nostoc sp. PCC 7120 under β-N-Methylamino-L-Alanine (BMAA) Treatment
Source: Toxins (Basel). 2020 Jun 4;12(6):372. doi: 10.3390/toxins12060372 (PMC7354497; doi:10.3390/toxins12060372)
Supplement: Supplementary file 1 [file toxins-12-00372-s001.zip › toxins-788877 supplementary for publish/toxins-788877 supplementary figures_tables for publish.docx]

Supplementary Materials: Proteomic Insights into Starvation of Nitrogen-Replete Cells of *Nostoc* sp. PCC 7120 under β-N-Methylamino-L-Alanine (BMAA) Treatment

Olga A. Koksharova, Ivan O. Butenko, Olga V. Pobeguts, Nina A. Safronova and
Vadim M. Govorun


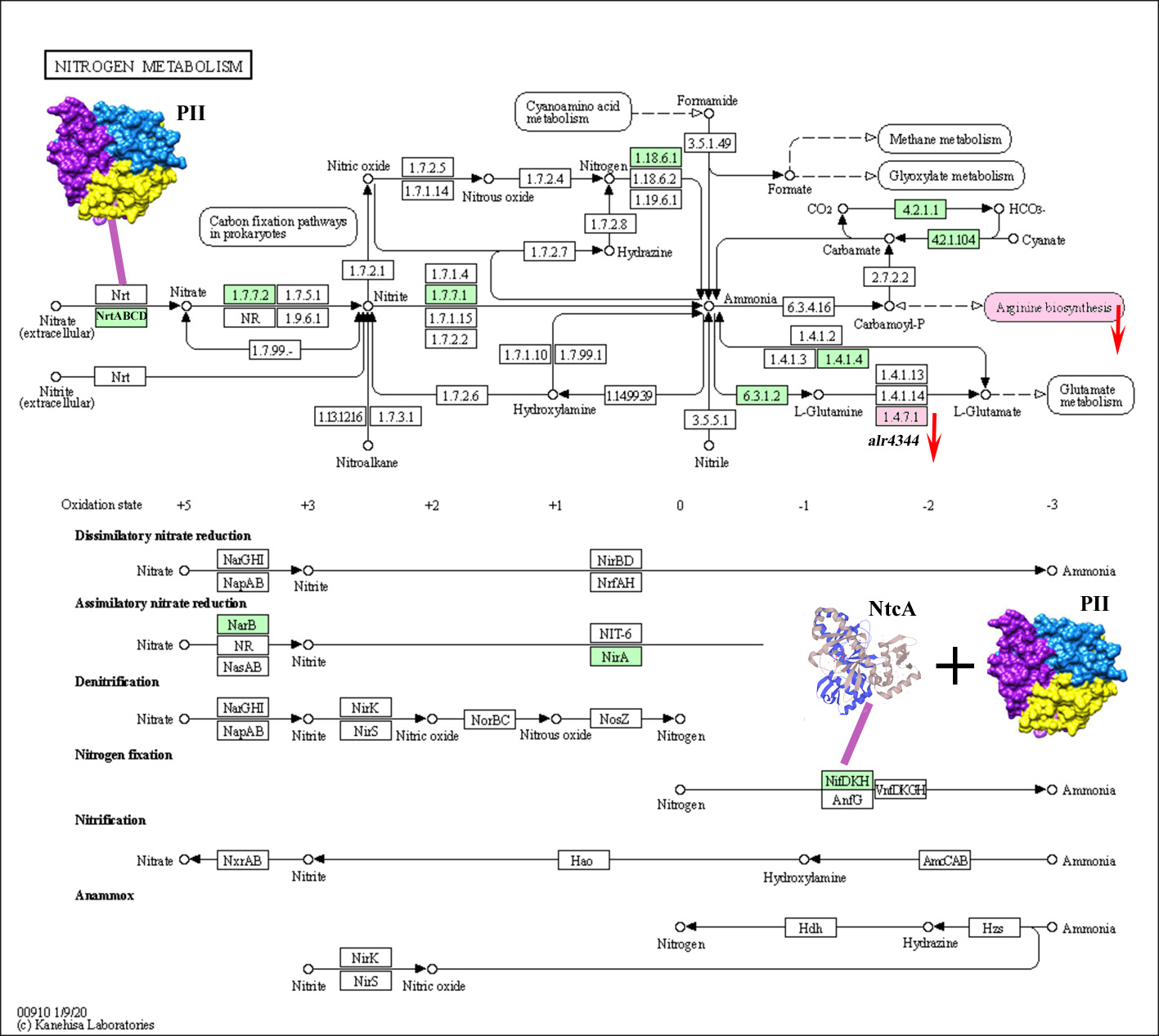


**Figure S1.** The impact of BMAA on nitrogen metabolism in nitrogen-replete cells of *Nostoc* 7120. The Nitrogen metabolism KEGG pathway map is presented (https://www.genome.jp/kegg-bin/show_pathway?ko00910+K00284). Glutamate synthase (ferredoxin) (EC:1.4.7.1, *alr4344*) is downregulated. Possible PII regulation is indicated.


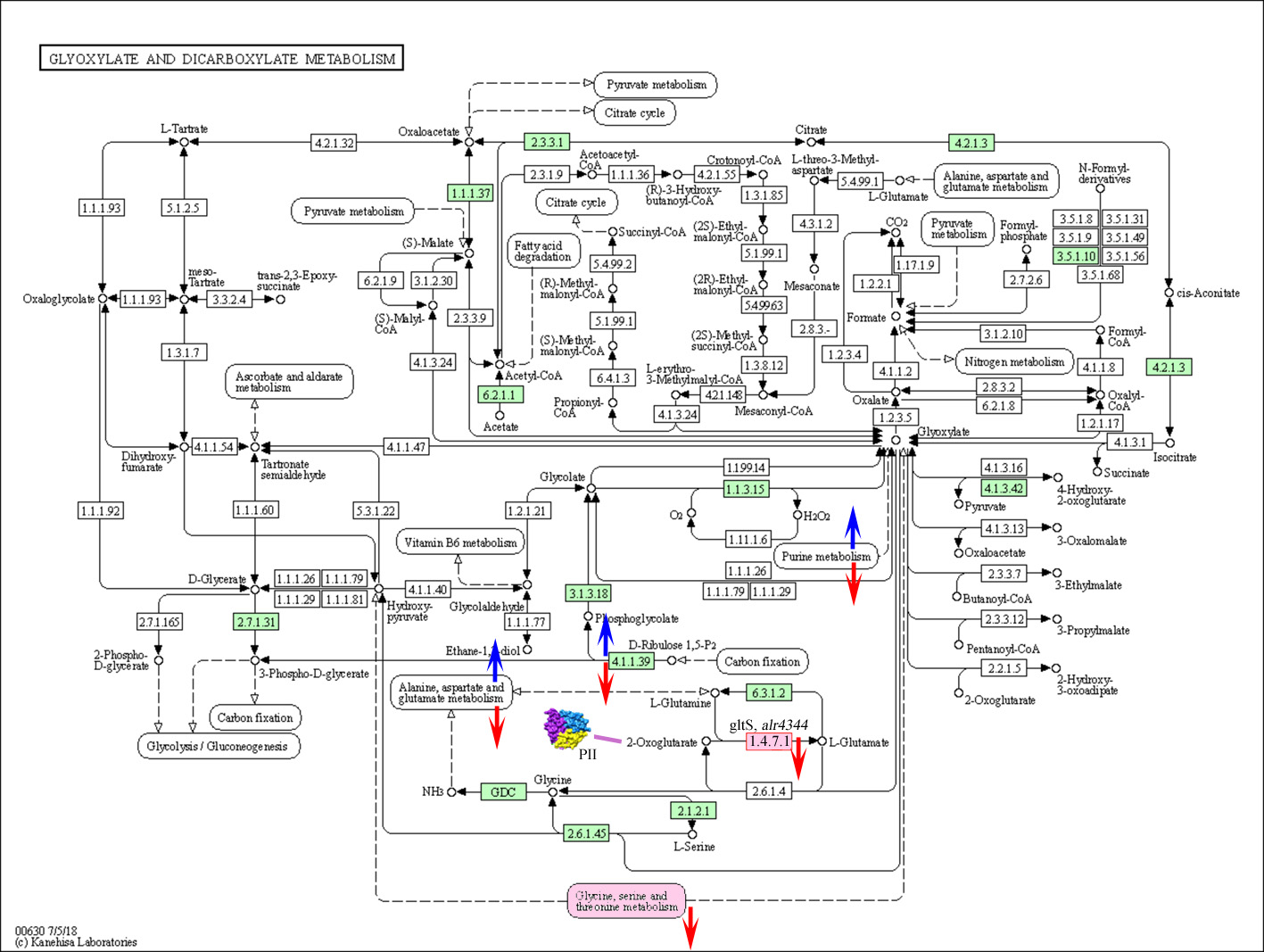


**Figure S2.** The BMAA impact on GltS (*alr4344*) in nitrogen-replete cells of *Nostoc* 7120. The Glyoxylate and dicarboxylate metabolism KEGG pathway map is presented (https://www.genome.jp/kegg-bin/show_pathway?ana00630+alr4344). Glutamate synthase (ferredoxin) [EC:1.4.7.1] is downregulated. Rubisco (4.1.1.39) subunits are regulated differently: RbcL is upregulated, while rbcS is downregulated (see also Supplementary Figure S4). Possible PII regulation is indicated. Pathways, in which identified in this study proteins participate, marked by arrows (blue—upregulation or red—downregulation).


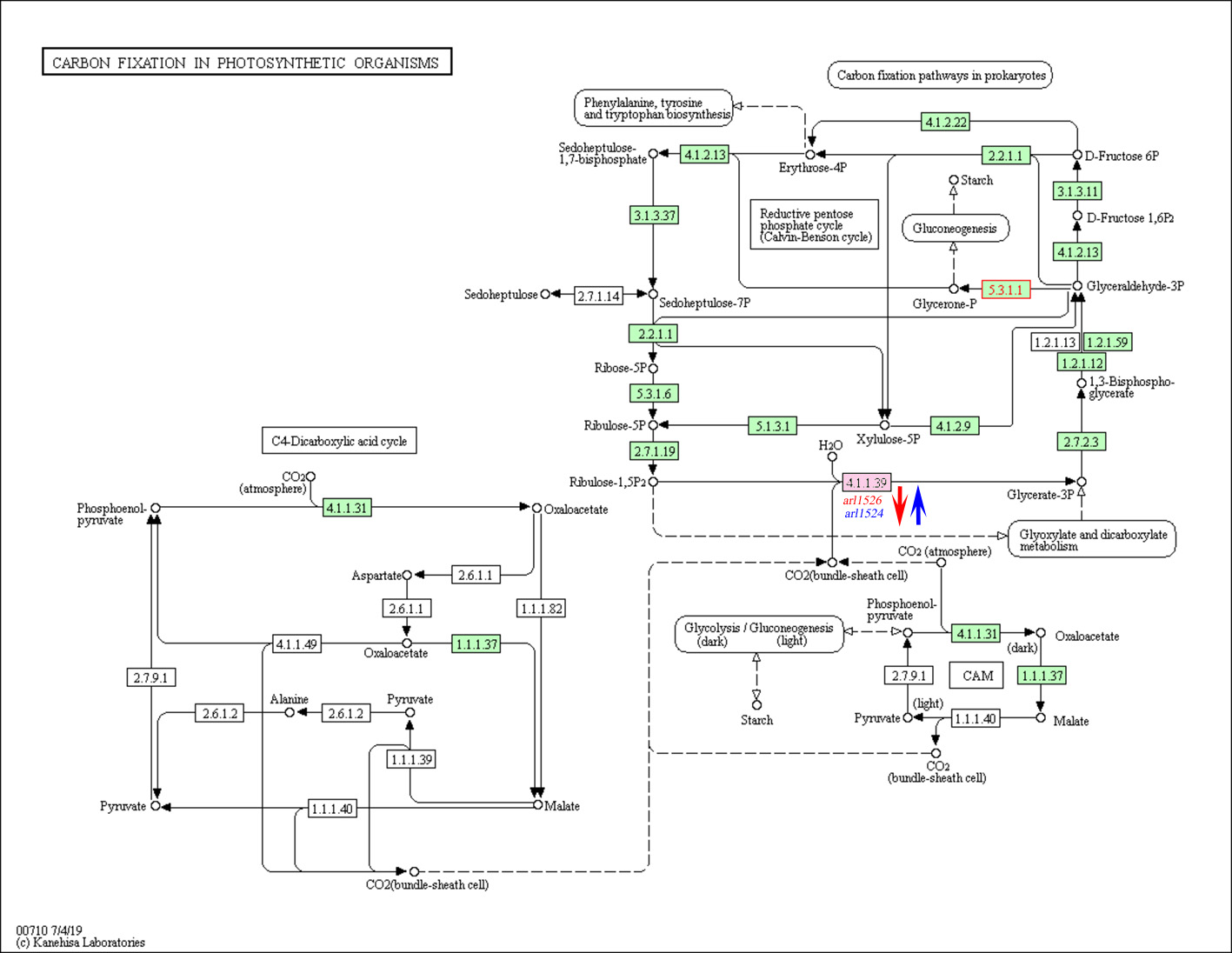


**Figure S3.** The BMAA impact on RbcL (*alr1524*) and on RbcS (*alr1526*) subunits of Rubisco in nitrogen-replete cells of *Nostoc* 7120. The Carbon fixation in photosynthetic organisms KEGG pathway map is presented (https://www.genome.jp/kegg-bin/show_pathway?ana00710+alr1524). RbcL is upregulated, while RbcS is downregulated. Identified in this study proteins, marked by arrows (blue—upregulation or red—downregulation).


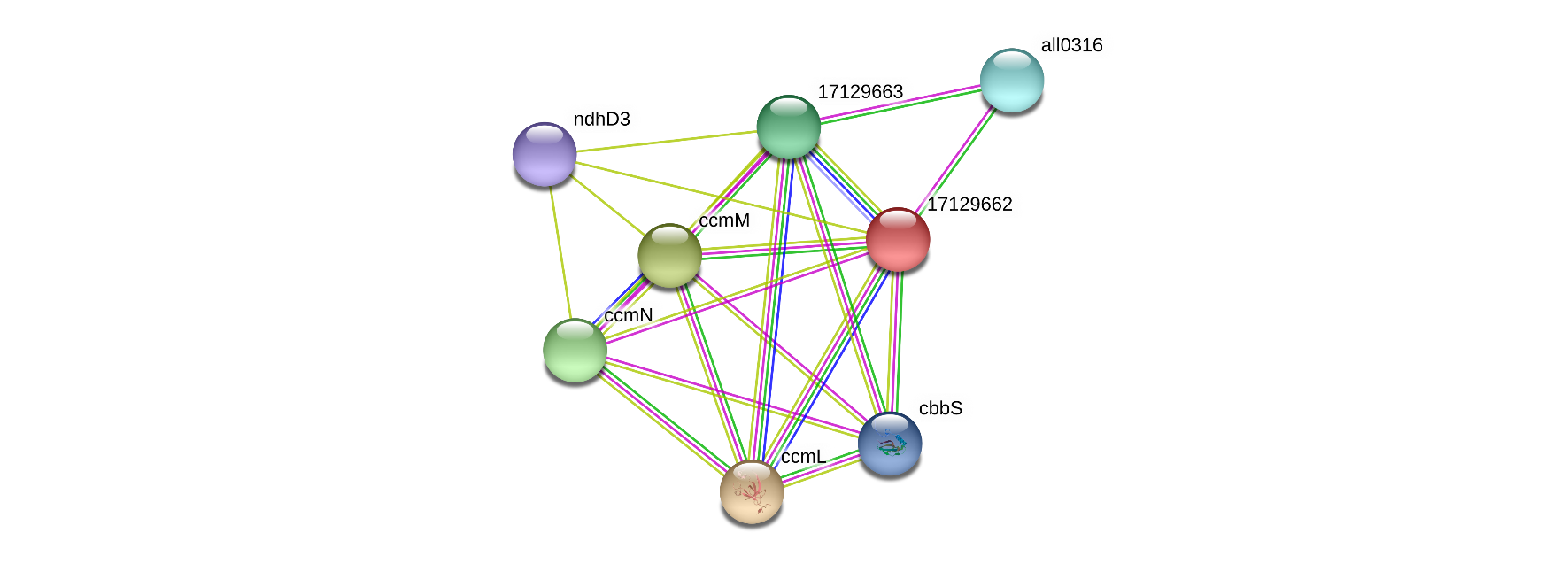


**Figure S4.** Protein network of ccmK and its protein partners. According to STRING (https://string-db.org), the ccmK protein ([*alr0317*](https://www.genome.jp/dbget-bin/www_bget?ana:alr0317)) (in the figure it is indicated as 17129662, red ball) interacts with Ribulose bisphosphate carboxylase smal chain (RbcS) (indicated as cbbS); ccmN, ccmM and ccmL are carboxisome proteins; 17129663 is ccmK ([*alr0318*](https://www.genome.jp/dbget-bin/www_bget?ana:alr0318)); ndhD3 is NAD(P)H-quinone oxidoreductase chain 4-3 (*alr5050*); all0316 is 2-hydroxy-6-oxohepta-2,4-dienoate.


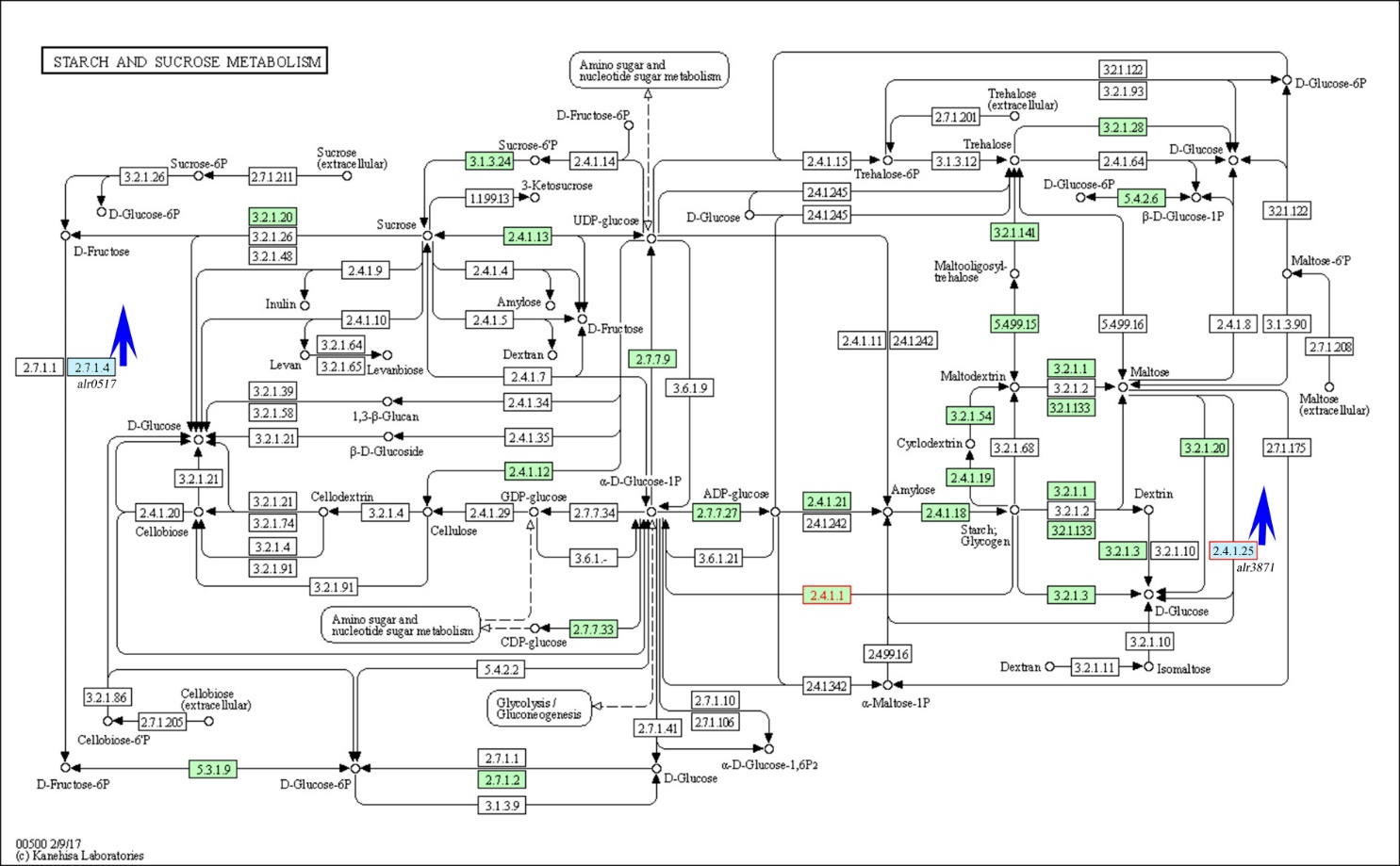


**Figure S5.** BMAA upregulates proteins involved in starch and sucrose metabolism of nitrogen-replete cells of *Nostoc* 7120. KEGG pathway map is presented the main enzymes involved in these pathways (https://www.genome.jp/kegg-bin/show_pathway?ana00500+alr3871). The enzymes 4-alpha-glucanotransferase [EC:2.4.1.25] (*alr3871*) and fructokinase [EC:[2.7.1.4](https://www.genome.jp/dbget-bin/www_bget?ec:2.7.1.4)] (*alr0517*) are upregulated (blue arrows).


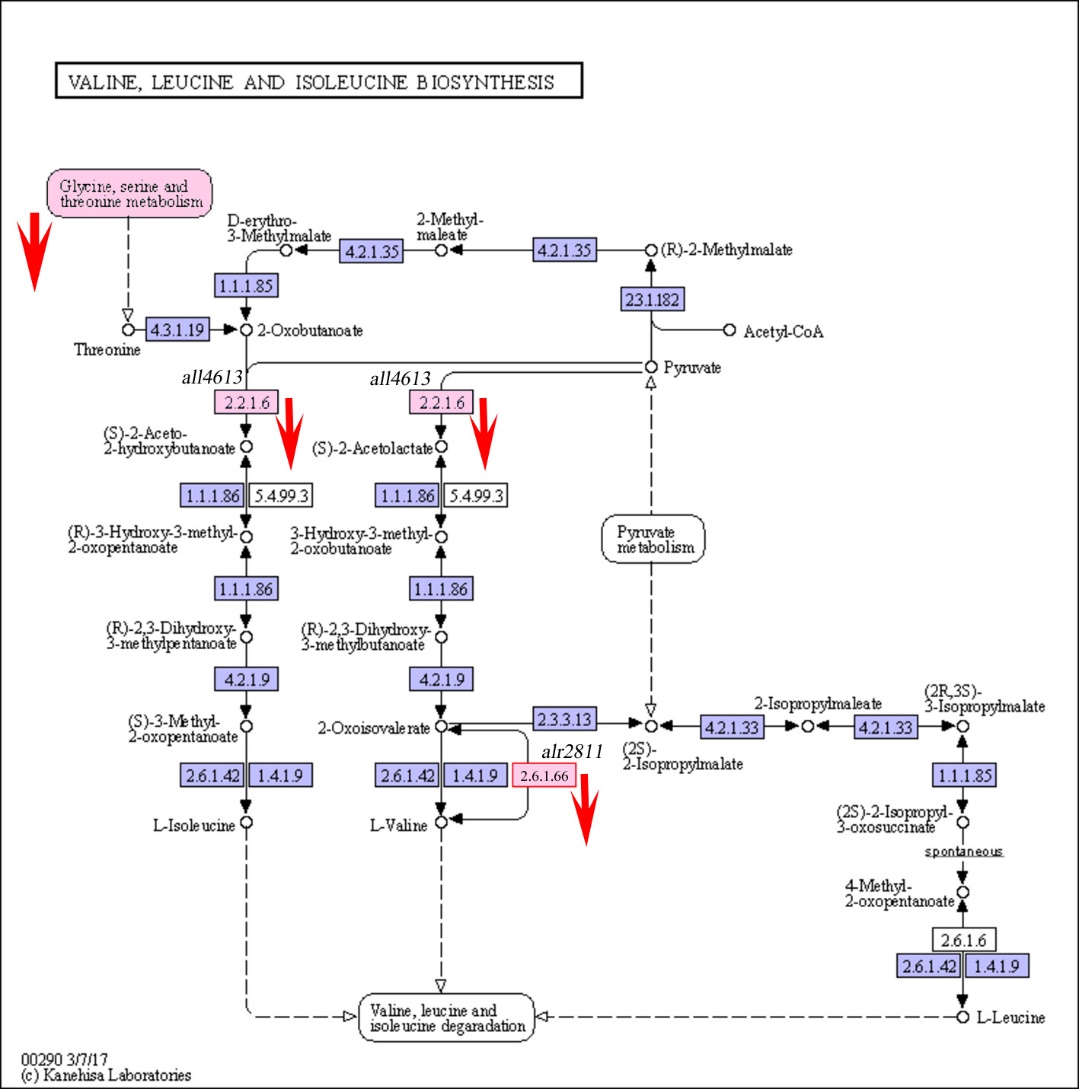


**Figure S6.** BMAA downregulates enzymes that are involved in valine, leucine and isoleucine biosynthesis (https://www.genome.jp/kegg-bin/show_pathway?ana00290+alr2811). Two enzymes, valine--pyruvate aminotransferase (EC:[2.6.1.66](https://www.genome.jp/dbget-bin/www_bget?ec:2.6.1.66), *alr2811*) and ilvG, acetolactate synthase I/II/III large subunit (EC:[2.2.1.6](https://www.genome.jp/dbget-bin/www_bget?ec:2.2.1.6), *all4613*) are downregulated.


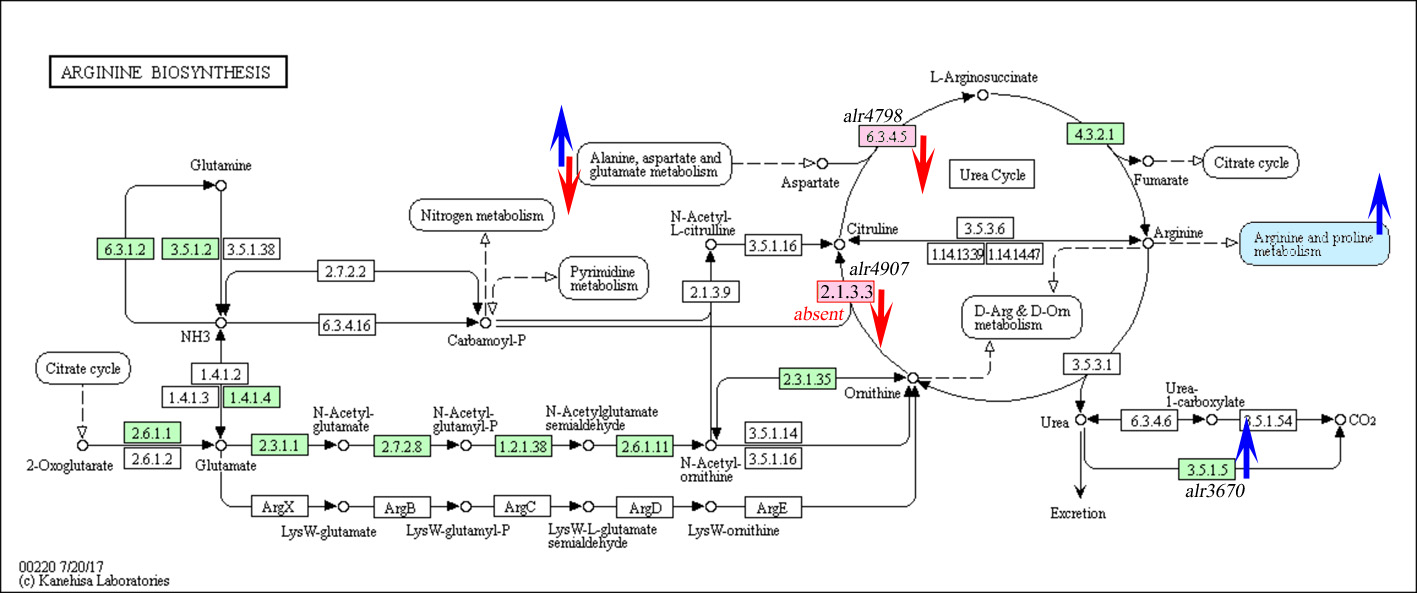


**Figure S7.** The impact of BMAA on arginine biosynthesis (<https://www.genome.jp/kegg-bin/show_pathway?ana00220+alr4907>). Argininosuccinate synthase (EC:6.3.4.5, *alr4798*) and ornithine carbamoyltransferase (EC:2.1.3.3, *alr4907*) are downregulated, while subunit alpha of urease (EC 3.5.1.5, *alr3670*) is slightly upregulated.


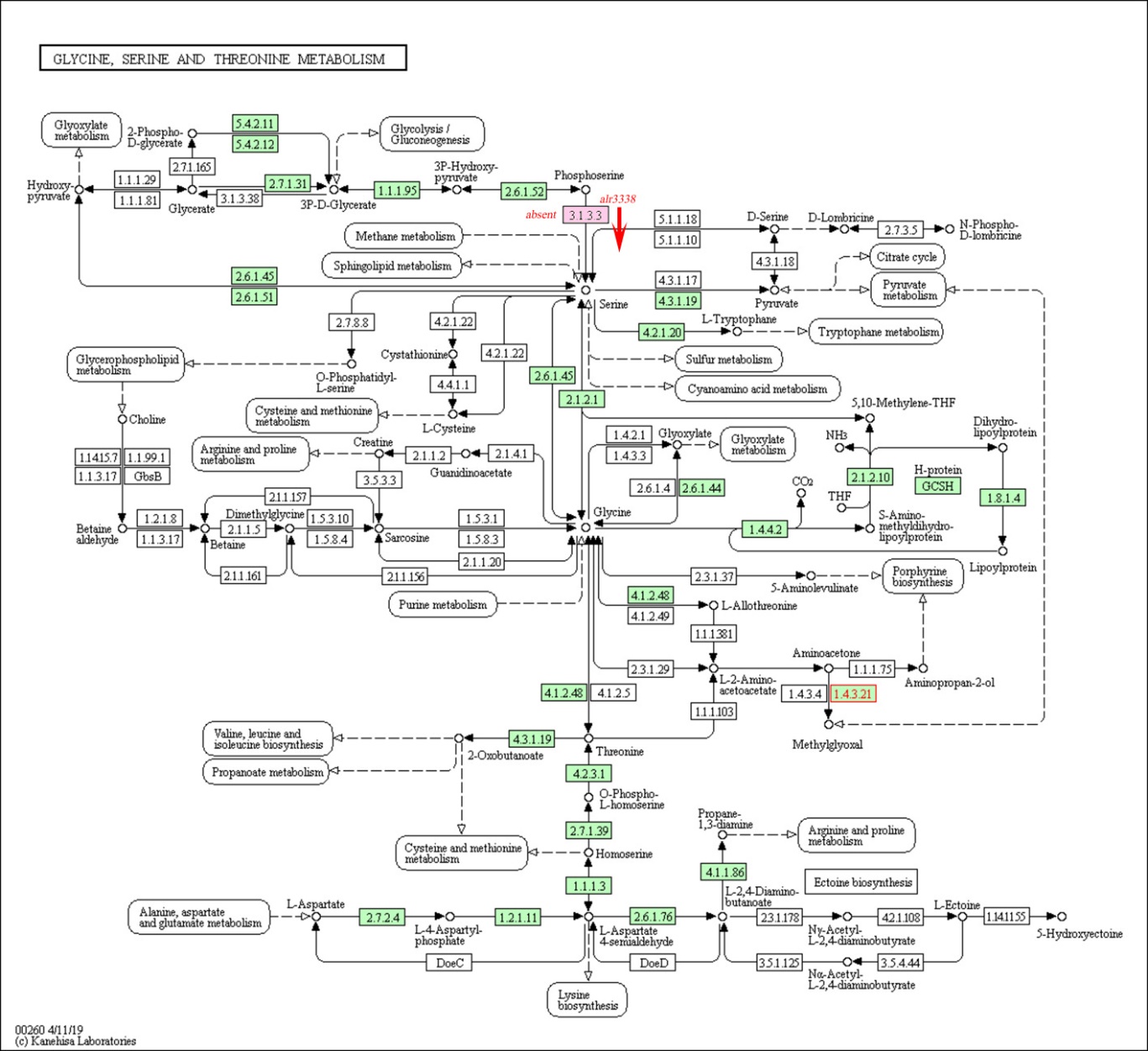


**Figure S8.** The impact of BMAA on glycine, serine and threonine metabolism (<https://www.genome.jp/kegg-bin/show_pathway?ana00260+alr3338>). The phosphoserine phosphatase (EC:3.1.3.3, *alr3338*) is absent in BMAA-treated cells of *Nostoc*.


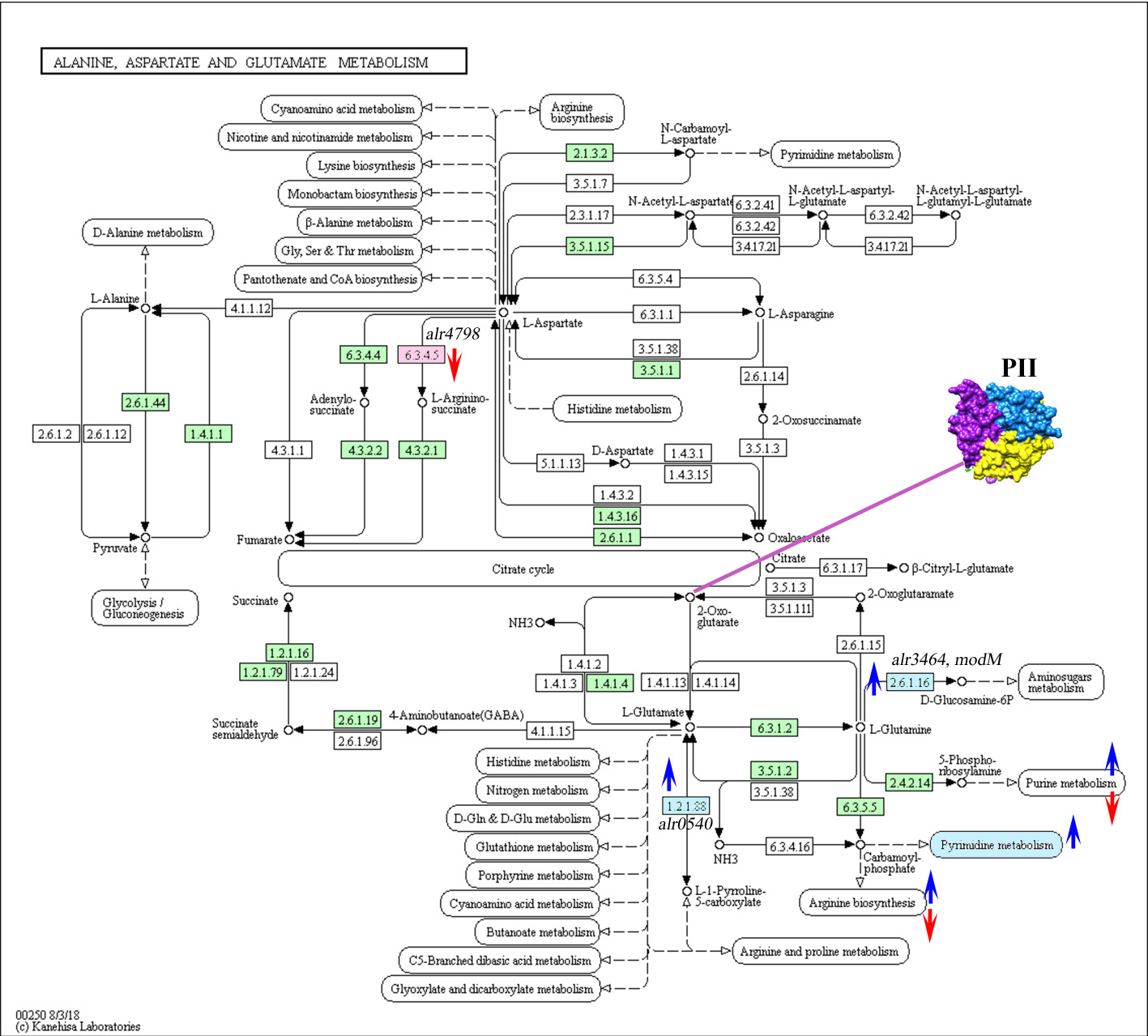


**Figure S9.** BMAA disturbs alanine, aspartate and glutamate metabolism pathways (<https://www.genome.jp/kegg-bin/show_pathway?ana00250+alr4798>). Two enzymes, glucosamine-fructose-6-phosphate aminotransferase (nodM, EC 2.6.1.16, *alr3464*) and 1-pyrroline-5 carboxylate dehydrogenase (EC 1.2.1.88, *alr0540*), are upregulated.


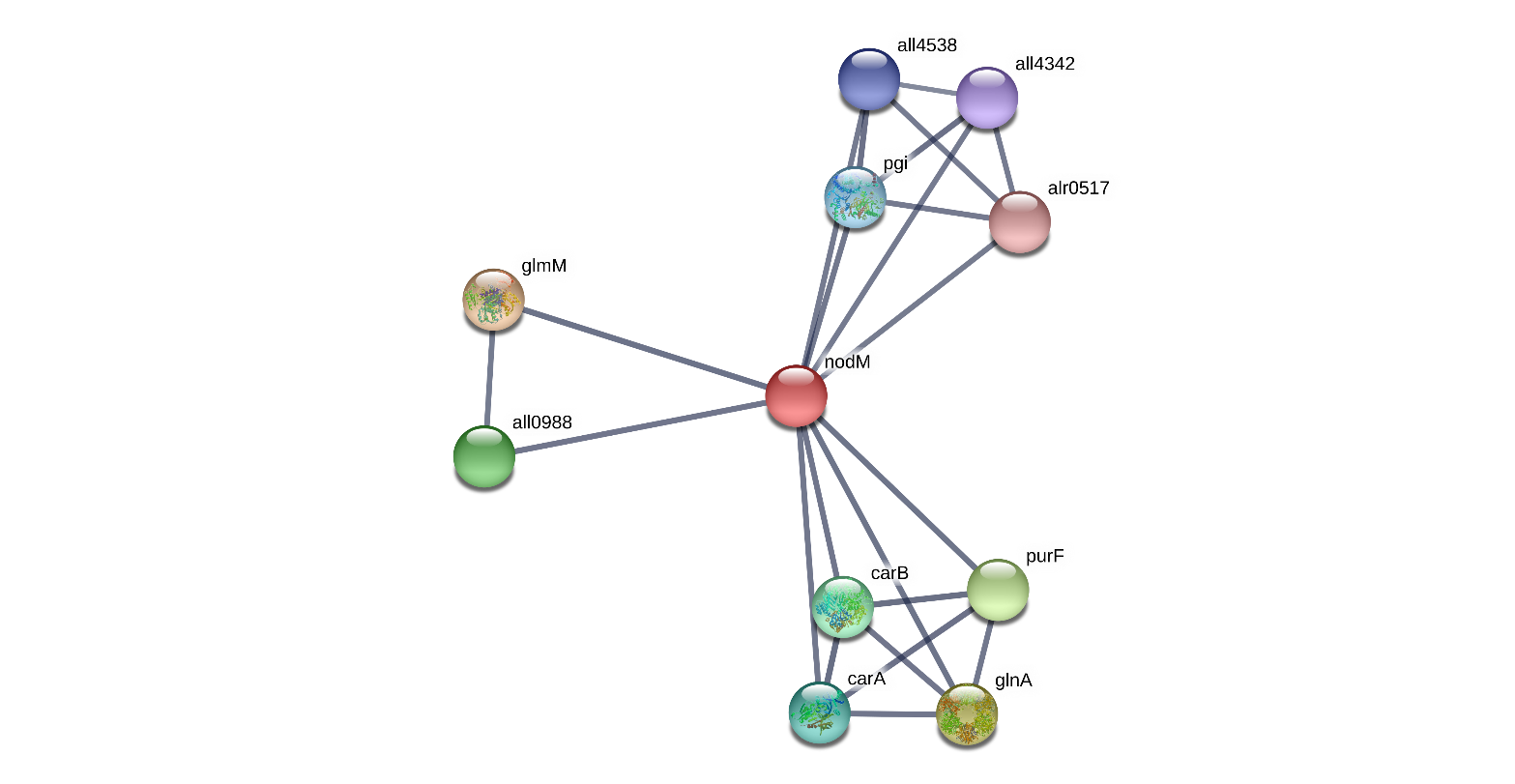


**Figure S10.** Protein network of glucosamine-fructose-6-phosphate aminotransferase (NodM, *alr3464*) and its protein partners, according to STRING (https://string-db.org), where glm M is phosphoglucosamine mutase that catalyzes the conversion of glucosamine-6-phosphate to glucosamine-1-phosphate; *all0988* is N-acetylglucosamine-6-phosphate deacetylase; glnA is glutamine synthetase that is involved in nitrogen metabolism via ammonium assimilation and catalyzes the ATP-dependent biosynthesis of glutamine from glutamate and ammonia; carA is carbamoyl-phosphate synthase small chain; carB is Carbamoyl-phosphate synthase large chain synthesizes [carbamoyl phosphate from bicarbonate](https://www.uniprot.org/uniprot/?query=organism:103690+pathway:402.68.68&sort=score) and involved in [L-arginine biosynthesis](https://www.uniprot.org/uniprot/?query=organism:103690+pathway:402.68&sort=score); purF is amidophosphoribosyltransferase that catalyzes the formation of phosphoribosylamine from phosphoribosylpyrophosphate (PRPP) and glutamine; *all4538* is mannose-6-phosphate isomerase; pgi is glucose-6-phosphate isomerase; *alr0517* is fructokinase; *all4342* is mannose-6-phosphate isomerase.

**Supplementary Table S2.** BMAA effect on hypothetical protein profile of *Nostoc* sp. PCC 7120 during growth in nitrogen-replete medium. The fold changes between the BMAA-treated samples and the control samples are shown, (p< 0.1).) (*) Proteins (genes) that are under transcriptional control of the nitrogen global regulator NtcA (according to CollecTF database).

| **No.** | **Gene** | **Up shifted** | **Down shifted** | ***p*-value** |
| --- | --- | --- | --- | --- |
| **Hypothetical Proteins (18 proteins)** | | | | |
| 1 | *alr4505** | 3.57 |  | 0.0931 |
| 2 | *alr0162* | 1.72 |  | 0.0677 |
| 3 | *All5250* | 2.08 |  | 0.0596 |
| 4 | *Alr1607* | 1.37 |  | 0.0563 |
| 5 | *all1353* | 1.09 |  | 0.0463 |
| 6 | *alr4359* | BTS* |  | 0.0344 |
| 7 | *all1411** | 2.7 |  | 0.0249 |
| 8 | *alr3297* | 1.29 |  | 0.0092 |
| 9 | *asr1156** | 1.89 |  | 0.0022 |
| 10 | *alr0740* | 1.72 |  | 0.0185 |
| 11 | *all1338* |  | Control** | 0.0017 |
| 12 | *asl4369* |  | Control | 0.00057 |
| 13 | *all4580* |  | Control | 0.0452 |
| 14 | *all7151* |  | 0.48 | 0.0002 |
| 15 | *alr5059* |  | 0.83 | 0.0695 |
| 16 | *all5091* |  | 0.70 | 0.0380 |
| 17 | *alr1133* |  | 0.39 | 0.0116 |
| 18 | *all4874* |  | 0.59 | 0.0045 |

* found only in BMAA treated sample; ** found only in control sample**Supplementary Table S3.** Gene coexpression data for identified hypothetical proteins in the proteome of *Nostoc* sp. PCC 7120 under BMAA treatment while growing in nitrogen-replete medium according to ALCOdbCyano (http://alcodb.jp/cyano/) are shown. The coexpressed genes encoding proteins that were identified in this study are marked by green.

| **Up-Shifted Hypothetical Proteins** | | | |
| --- | --- | --- | --- |
| **No.** | **Hypothetical Gene** | **Pair Similar Regulated Genes and Proteins, Identified in This Study** | **List of Some Coexpressed Genes**  **from the ALCOdbCyano Database** |
| 1 | *alr4505* |  | [*alr2991*](http://alcodb.jp/cyano/PCC7120/alr2991/list) Dna J protein |
| 2 | *alr0162* |  | [*alr5182*](http://alcodb.jp/cyano/PCC7120/alr5182/list) Oxidoreductase  [*alr0072*](http://alcodb.jp/cyano/PCC7120/alr0072/list) Two-component response regulator |
| 3 | *all5250* |  | *all4750* Two-component response regulator |
| 4 | *alr1607* |  | [*alr3655*](http://alcodb.jp/cyano/PCC7120/alr3655/list) Photomixotrophic growth related protein, PmgA homolog |
| 5 | *all1353*  Putative hydrolase |  | [*alr3655*](http://alcodb.jp/cyano/PCC7120/alr3655/list) Photomixotrophic growth related protein, PmgA homolog |
| 6 | *alr4359* | *alr4359-*[*alr2372*](http://alcodb.jp/cyano/PCC7120/alr2372/list) | [alr5084](http://alcodb.jp/cyano/PCC7120/alr5084/list) Endopeptidase Clp ATP-binding chain B  [all4936](http://alcodb.jp/cyano/PCC7120/all4936/list) Cell division protein FtsH  [alr2372](http://alcodb.jp/cyano/PCC7120/alr2372/list) ABC transporter ATP-binding protein |
| 7 | *all1411* | *all1411- all0868* | [all1588](http://alcodb.jp/cyano/PCC7120/all1588/list) 33kD chaperonin, heat shock protein HSP33  [all1173](http://alcodb.jp/cyano/PCC7120/all1173/list) DNA-binding protein, starvation-inducible  [all0860](http://alcodb.jp/cyano/PCC7120/all0860/list) DNA gyrase A subunit  [all0863](http://alcodb.jp/cyano/PCC7120/all0863/list) Carbon dioxide concentrating mechanism protein CcmK  all1759 Cell division protein FtsY |
| 8 | *alr3297* |  | [alr3421](http://alcodb.jp/cyano/PCC7120/alr3421/list) Plastoquinol--plastocyanin reductase, cytochrome b6; PetB  [asr1592](http://alcodb.jp/cyano/PCC7120/asr1592/list) 30S ribosomal protein S20  [alr4812](http://alcodb.jp/cyano/PCC7120/alr4812/list) Heterocyst differentiation related protein PatN |
| 9 | *asr1156* | *asr1156-* asr3935 | [asr4775](http://alcodb.jp/cyano/PCC7120/asr4775/list) Photosystem I subunit X  asr3935 DNA binding protein HU  [asr3463](http://alcodb.jp/cyano/PCC7120/asr3463/list) Photosystem I iron-sulfur protein PsaC  [alr2818](http://alcodb.jp/cyano/PCC7120/alr2818/list) Heterocyst differentiation protein HetP  [alr4392](http://alcodb.jp/cyano/PCC7120/alr4392/list) Nitrogen-responsive regulatory protein NtcA |
| **Down-Shifted Hypothetical Proteins** | | | |
| 1 | *all1338* |  | [*all1291*](http://alcodb.jp/cyano/PCC7120/all1291/list) Cyanate lyase; CynS  [*all1368*](http://alcodb.jp/cyano/PCC7120/all1368/list) Amidotransferase  [*all1101*](http://alcodb.jp/cyano/PCC7120/all1101/list) Ferrichrome iron receptor  [*all1304*](http://alcodb.jp/cyano/PCC7120/all1304/list) Sulfate permease family protein |
| 2 | *asl4369* |  | [*asl0009*](http://alcodb.jp/cyano/PCC7120/asl0009/list) ATP synthase subunit c; AtpH  [*alr5297*](http://alcodb.jp/cyano/PCC7120/alr5297/list) 50S ribosomal protein L19 |
| 3 | *all4580* |  | [all4575](http://alcodb.jp/cyano/PCC7120/all4575/list) Phosphate ABC transporter, phosphate-binding periplasmic protein |
| 4 | *all7151* |  | There are no matches to this query |
| 5 | *alr5059* | *alr5059-*[*alr0537*](http://alcodb.jp/cyano/PCC7120/alr0537/list) | [*alr1545*](http://alcodb.jp/cyano/PCC7120/alr1545/list) Steroid delta-5-3-ketosteroid isomerase  [*alr0537*](http://alcodb.jp/cyano/PCC7120/alr0537/list) Phycobilisome rod-core linker protein cpcG  [*asl0009*](http://alcodb.jp/cyano/PCC7120/asl0009/list) ATP synthase subunit c; AtpH |
| 6 | *all5091* |  | [all4148](http://alcodb.jp/cyano/PCC7120/all4148/list) Ferredoxin I  [all4121](http://alcodb.jp/cyano/PCC7120/all4121/list) Ferredoxin--NADP(+) reductase |
| 7 | *alr1133* | *alr1133- alr1073* | [asl0749](http://alcodb.jp/cyano/PCC7120/asl0749/list) 30S ribosomal protein S15  all4501 Phosphate regulon transcriptional regulator  alr1073 Isoleucyl-tRNA synthetase; IleS |
| 8 | *all4874* |  | [all4822](http://alcodb.jp/cyano/PCC7120/all4822/list) Similar to beta-lactamase  [alr3858](http://alcodb.jp/cyano/PCC7120/alr3858/list) Cell division protein FtsZ  [all5039](http://alcodb.jp/cyano/PCC7120/all5039/list) ATP synthase beta subunit  [alr5065](http://alcodb.jp/cyano/PCC7120/alr5065/list) UDP-N-acetylmuramoylalanine--D-glutamate ligase |
